# Light-Inducible System for Tunable Protein Expression in *Neurospora crassa*

**DOI:** 10.1534/g3.112.003939

**Published:** 2012-10-01

**Authors:** Jennifer M. Hurley, Chen-Hui Chen, Jennifer J. Loros, Jay C. Dunlap

**Affiliations:** * Department of Genetics and; † Department of Biochemistry, Geisel School of Medicine at Dartmouth, Hanover, New Hampshire 03755

## Abstract

Filamentous fungi are important model systems for understanding eukaryotic cellular processes, including the study of protein expression. A salient feature of fungi is the ability of the protein-processing machinery to perform all of the extensive posttranslational modifications needed in the complex world of eukaryotic organisms, making them great hosts for production of eukaryotic proteins. In the model organism *Neurospora crassa*, several regulatable promoters have been used for heterologous gene expression but all suffer from leaky expression absent stimuli or an inability to induce protein expression at levels greater than those seen *in vivo*. To increase and better control *in vivo* protein expression in Neurospora, we have harnessed the light-induced *vvd* promoter. *vvd* promoter-driven mRNA expression is dependent upon light, shows a graded response, and is rapidly shut off when returned to the dark. The *vvd* promoter is a highly tunable and regulatable system, which could be a useful instrument for those interested in efficient and controllable gene expression.

Light, beyond being the energy source for much of the world, is the primary cue for many biological processes. A powerful transcriptional response to light is seen in the filamentous fungus *Neurospora crassa* (Bahn *et al.* 2007; Chen *et al.* 2010b; Corrochano 2007; Heintzen and Liu 2007; Herrera-Estrella and Horwitz 2007; Liu and Bell-Pedersen 2006), and for this reason Neurospora has served as a model system for studying the effects of light on higher eukaryotes for many years (Chen and Loros 2009). Neurospora has two known blue light receptors, White Collar-1 (WC-1) and Vivid (VVD) (Froehlich *et al.* 2002; He *et al.* 2002; Heintzen *et al.* 2001; Schwerdtfeger and Linden 2003). WC-1 forms a complex with the transcriptional coactivator White Collar-2 (WC-2), termed the White Collar Complex (WCC) (Ballario and Macino 1997; Froehlich *et al.* 2002). Upon activation by light, the WCC binds to and activates promoters of light-responsive genes, one of which is *vvd* (Ballario and Macino 1997; Chen *et al.* 2009; Heintzen *et al.* 2001). Binding of the WCC to the *vvd* promoter leads to the expression of VVD and in turn VVD interacts directly with the WCC to negatively regulate the light response initiated by the WCC (Chen *et al.* 2010a; Hunt *et al.* 2010; Malzahn *et al.* 2010). Through this mechanism, VVD plays a large role in Neurospora’s ability to adapt to various levels of light intensity (Malzahn *et al.* 2010; Schwerdtfeger and Linden 2003). Through its function as a regulator of the WCC, VVD also plays roles in entrainment of the clock, including gating of light input and temperature compensation of phase (Elvin *et al.* 2005; Heintzen *et al.* 2001; Hunt *et al.* 2007).

*vvd* is only very weakly expressed in the dark but when activated by light, *vvd* is rapidly and transiently induced by as much as 300-fold within minutes of light induction (Elvin *et al.* 2005; Heintzen *et al.* 2001). WCC-driven induction of transcriptional repressors such as CSP-1 as well as VVD inhibition of WCC activity leads to repression of *vvd* transcription within 60 min of exposure to light (Chen *et al.* 2010a; Heintzen *et al.* 2001; Hunt *et al.* 2010; Malzahn *et al.* 2010). We theorized because of these properties we could use the *vvd* promoter as a tool to drive expression of other endogenous Neurospora proteins as well as exogenous proteins and moreover, by expressing our gene of interest in a *vvd* knockout background, we could further increase expression from the *vvd* promoter (Heintzen *et al.* 2001).

As proof of principle, we expressed three different genes under the regulation of the *vvd* promoter, *wc-1*, *gfp* (encoding green fluorescent protein) (Freitag *et al.* 2004), and *gh5-1* (encoding a cellulase used in biofuel production) (Sun *et al.* 2011), all to between 50-fold and 150-fold the uninduced level. The heterologous *gfp* gene was driven at levels similar to the native *wc-1* and *gh5-1* genes, showing a graded response to light duration and intensity and rapidly returning to the inactive state after the light pulse. Finally, the *vvd* promoter produced three-fold more protein than the *ccg-1* promoter that is commonly used for constitutive overexpression. Using the *vvd* promoter, we have created a novel tunable and controllable system to express both endogenous and exogenous genes in *N. crassa*.

## Materials and Methods

### Strains

The wild-type (WT) strain used was OR74A whereas the *Δvvd* strain FGSC11556 came from the Neurospora knockout project (Colot *et al.* 2006). Transformations were performed as described (Bardiya and Shiu 2007; Colot *et al.* 2006) and screened by allelic-specific nested polymerase chain reaction (PCR; primers in supporting information, Table S1). *gh5-1-gfp-6xhis* driven by the *ccg*-1 promoter was a generous gift from L. Glass (Sun *et al.* 2011). *vvd*::*hph+*; *Δcsr*::*pvvd3000bp*::*gh5-1*::*gfp*::*his^6^* was created by transforming pVVD-GH-5 (Figure S1) into FGSC11556; *Δcsr*::*pvvd3000bp*::*wc-1*::*v^5^*::*bar* was created by transforming pVVD-WC-1 (Figure S1) into OR74A; *Δvvd*::*hph+*; *Δcsr*::*pvvd3000bp*::*wc-1*::*v^5^*::*bar* was created by transforming pVVD-WC-1 (Figure S1) into FGSC11556; *Δvvd*::*hph+*; *Δcsr*::*pvvd3000bp*::*gfp*::*bar* was created by transforming pVVD-GFP (Figure S1) into FGSC11556. The pVVD-GFP has been deposited into the FGSC, where FGSC11556 and other pertinent strains are already available.

### Culture conditions and light treatment

Light treatment was performed as described previously with some differences (Chen *et al.* 2009). A total of 1 × 10^7^ of 1-week-old conidia were inoculated into a 10-cm Petri dish with 20 mL of Bird medium (Metzenberg 2004) containing 1.8% glucose (for the *vvd* promoter) or Vogel’s salts with 2% glucose (for the *ccg-1* promoter). After 24 hr of incubation in darkness at 25°, a mycelia plug was cut with a No. 6 cork borer (12-mm diameter) and transferred into a 125-mL flask with 50 mL of Bird medium containing 1.8% glucose. All procedures stated as in darkness (DD) were performed under a low-intensity red light environment to avoid any possible light-stimulating effects (Aronson *et al.* 1994). After another 24 hr of culture with constant shaking (125 rpm) in DD at 25°, the flasks were moved to a shaker at 25° with a continuous white light stimulus (LL), covering a wide range of the spectrum from 400 to 700 nm (cool white fluorescent light bulb; General Electric F20T12-CW, modifiable from 0.01 to 25 μeinsteins·m^2^·s^−1^), and then harvested before (DD) and from 1 (LL 1), 2 (LL 2), 4 (LL 4), 8 (LL 8), 24 (LL 24), 32 (LL 32) up to 48 (LL 48) hr of white light treatment. In the case of the *ccg-1* promoter, this light shift was accompanied by a shift from Vogel’s salts with 2% glucose to Vogel’s salts with 2% sodium acetate (Vogel 1956). Using vacuum filtration, we harvested mycelia, which were immediately frozen in liquid nitrogen and stored at −80° until RNA or protein extraction.

### Western blots and protein preparation

Protein lysates and Western blot analysis were performed as described (Chen *et al.* 2010a) with slight modifications. Protein lysates were prepared on a small scale with a protease inhibitor mixture (P9599; Sigma-Aldrich). For Western blot analysis, 10 μg of total protein was loaded per lane. Anti-V5 antibody (Invitrogen) was diluted 1:5000. Anti-GFP antibody (sc-9996; Santa Cruz Biotechnology) was diluted 1:200. SuperSignal West Femto ECL (Pierce) was used for signal development. GFP standard was from Clontech (cat. no. 632373).

### RNA extraction, cDNA preparation, and quantitative PCR (qPCR)

RNA extractions were performed as described previously with the following modifications (Chen *et al.* 2009). Total RNA was extracted by adding 1 mL of TRIZOL reagent (Invitrogen) to the frozen tissue and then pulverizing it using a homogenizer (Tissuelyzer; QIAGEN) containing stainless steel beads (5 mm). The tissue was separated from the supernatant using rapid centrifugation (16,000 × *g* for 15 min) and further purified using chloroform extraction and ethanol precipitation. After quantification by a spectrophotometer, 2 μg of RNA from each time point was used for cDNA synthesis using the Superscript III first-strand synthesis system for RT-PCR (Invitrogen) as described (Chen *et al.* 2009). Analysis of cDNA was performed using the SYBR green-based method (ABI) with the primer sets summarized in (Table S1). RT-qPCR data were measured as described previously (Chen *et al.* 2010a).

## Results and Discussion

### *gfp* demonstrates the ability of the *vvd* promoter to express exogenous genes

**Figure 1:**
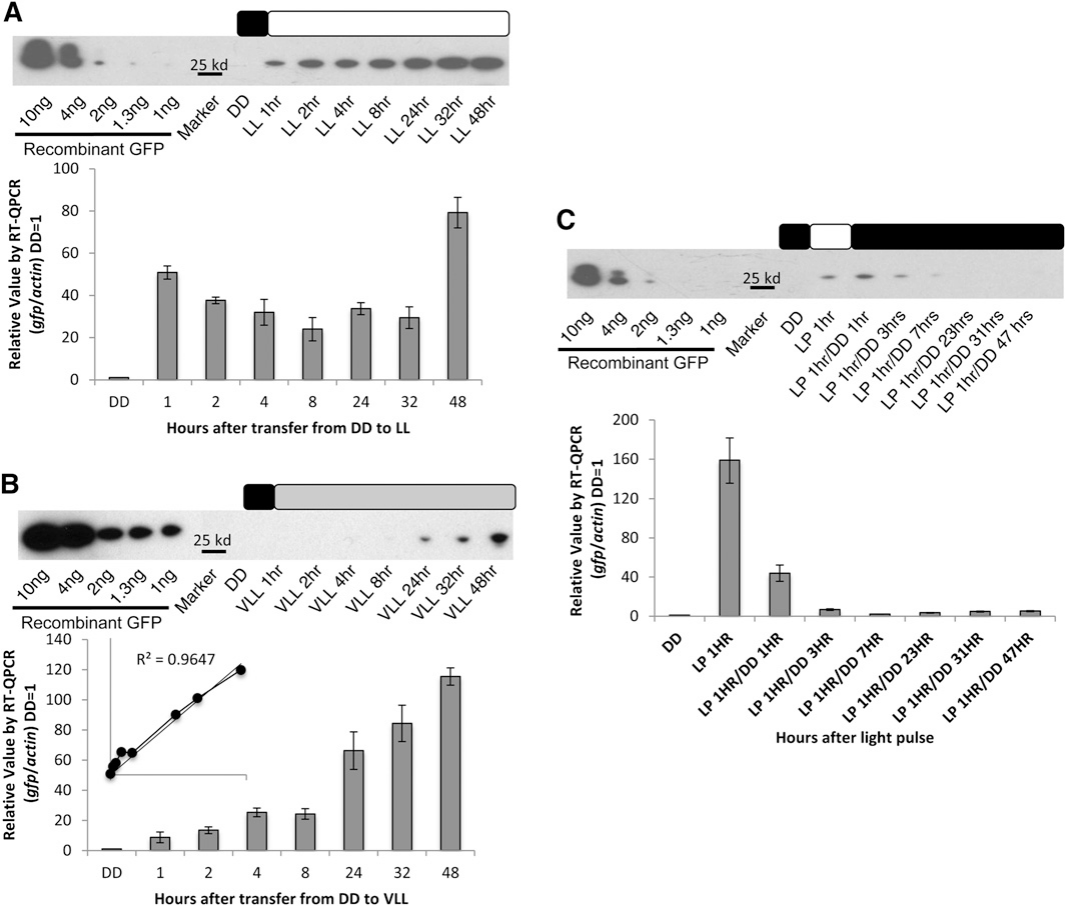
Tunable expression driven by the *vvd* promoter. Expression from the *vvd* promoter was determined at the transcriptional and translational levels in the *Δvvd* background by measuring induction of *gfp* at (A) high light (LL), (B) very low light (VLL), and (C) light exposure for h hr at LL, using RT-qPCR analysis compared with actin (*n* = 3, error bars represent ± 1 SE) and western blot analysis compared with a GFP standard. The inset in panel B confirms that *gfp* expression increases linearly with light exposure. Boxes describe the light regime; black is lights off, white is lights on, and gray is lights on to very low light levels. DD is dark conditions, LL is 25 μeinsteins·m^2^·s, VLL is 0.01 μeinsteins·m^2^·s, and LP is LL for 1 hr.

We began by demonstrating using GFP that the *vvd* promoter was capable of inducing expression of exogenous genes (Chiu *et al.* 1996; Corish and Tyler-Smith 1999; de Ruijter *et al.* 2003; Freitag *et al.* 2001). After growing a *Δvvd* strain bearing *pvvd-gfp* in DD for 48 hr, *gfp* mRNA and GFP protein levels were tracked with RT-qPCR and western blot analysis respectively after the strain was shifted to LL. *gfp* mRNA expression was induced 60-fold over background levels after DD to LL transfer, increasing within an hour to more than 50-fold and to more than 80-fold after 48 hr. At the protein level, there was no GFP detected at the DD time point, and GFP levels increased steadily over the entire 48-hr period to between 0.2 and 0.4 ηg/μL (in 10 mg of total protein) of prepared protein (Figure 1A).

### The *vvd* promoter is capable of tunable expression and rapid responses

One hallmark of an effective promoter is that expression levels can be tuned by the inducer, as in the case of the *qa*-2 promoter (Aronson *et al.* 1994; Shi *et al.* 2010). To evaluate the tunability of the *vvd* promoter, we exposed the GFP strain to 25 μeinsteins·m^2^·s, 0.25 μeinsteins·m^2^·s, and 0.01 μeinsteins·m^2^·s of white light. Although 25 and 0.25 μeinsteins·m^2^·s appeared to drive similar amounts of *gfp* expression (Figure 1A and data not shown), 0.01 μeinsteins·m^2^·s yielded distinctly lower expression of *gfp* mRNA (Figure 1B). In 1 hr of exposure to 0.01 μeinsteins·m^2^·s of light, there was only a 10-fold increase in induction of *gfp* mRNA (Figure 1B) as compared with 60-fold under 25 μeinsteins·m^2^·s of light (Figure 1A). Previous work has shown that *vvd* is induced by a few minutes of light (Heintzen *et al.* 2001), and here GFP protein expression (Figure 1B) increased in direct proportion to light exposure during the 48-hr period (R^2^ = 0.9647).

Because the *vvd* promoter is tightly regulated in the dark (Schwerdtfeger and Linden 2003), we expected that shifting the culture back to DD would turn off expression of the gene of interest. The GFP strain was exposed for 1 hr to 25 μeinsteins·m^2^·s of white light, returned to DD, and the levels of protein and mRNA were tracked from 1 to 48 hr. After the anticipated light-induced increase (Figure 1, A and C) there was a rapid decrease in activity at the promoter reflecting a decay constant at the promoter of ~20 min such that the promoter had returned to its inactive state by 3 hr after light pulse, and protein levels near zero, by 8 hr after the light pulse (Figure 1C).

### Expression of the *wc-1* gene under the *vvd* promoter demonstrates increased expression in the *Δvvd* strain compared with WT

**Figure 2:**
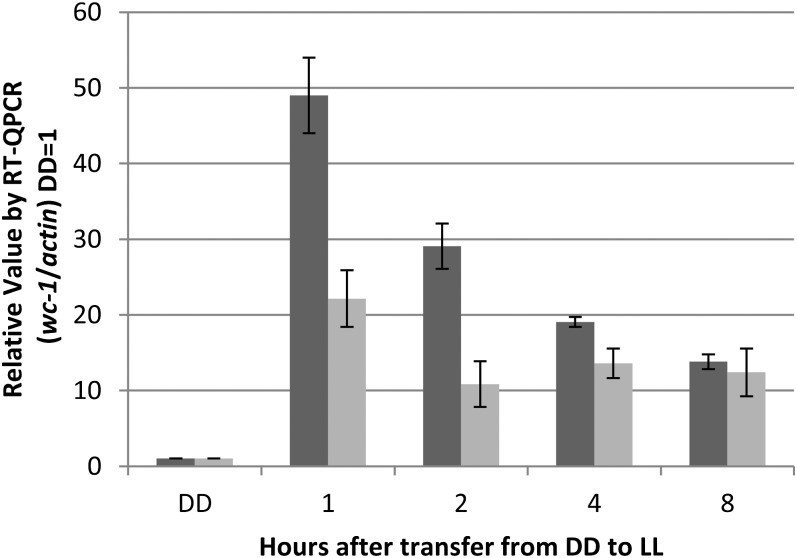
Expression of *wc*-1 driven by the *vvd* promoter in the *Δvvd* background is greater than in WT. DD is dark conditions, and LL is 25 μeinsteins·m^2^·s. Transcriptional levels were determined measuring induction of *wc-1^v5^* in WT (light gray) and *Δvvd* (dark gray) strains using RT-qPCR analysis compared with *actin* (*n* = 3, error bars represent ± SE).

To confirm that the *vvd* promoter is capable of driving expression at a greater level in the *Δvvd* strain as compared with the WT strain, we grew WT and Δ*vvd* strains bearing a V5-tagged *wc-1* driven by the *vvd* promoter in the dark (DD) for 48 hr before exposing the cultures to white light (LL; 25 μeinsteins·m^2^·s) for 8 hr. mRNA levels were tracked using RT-qPCR using primers targeted to the V5 tag region; thus, the only gene tracked was *wc-1^V5^*. Upon transfer to light, a rapid and distinct increase in transcript expression was seen in both the WT and *Δvvd* strains, and high expression was sustained for the duration of the experiment (Figure 2). Compared with *wc-1* mRNA levels driven by the *wc-1*−native promoter (induced approximately 5-fold) (Lee *et al.* 2003), we observed a 50-fold induction of *wc-1* mRNA over background. Interestingly, the *Δvvd* strain showed 2- to 3-fold greater expression compared with the WT strain, confirming that removal of *vvd* from the genome alleviates repression of active WC-1.

### Expression levels were greater under the *vvd* promoter as compared with the *ccg-1* promoter

**Figure 3:**
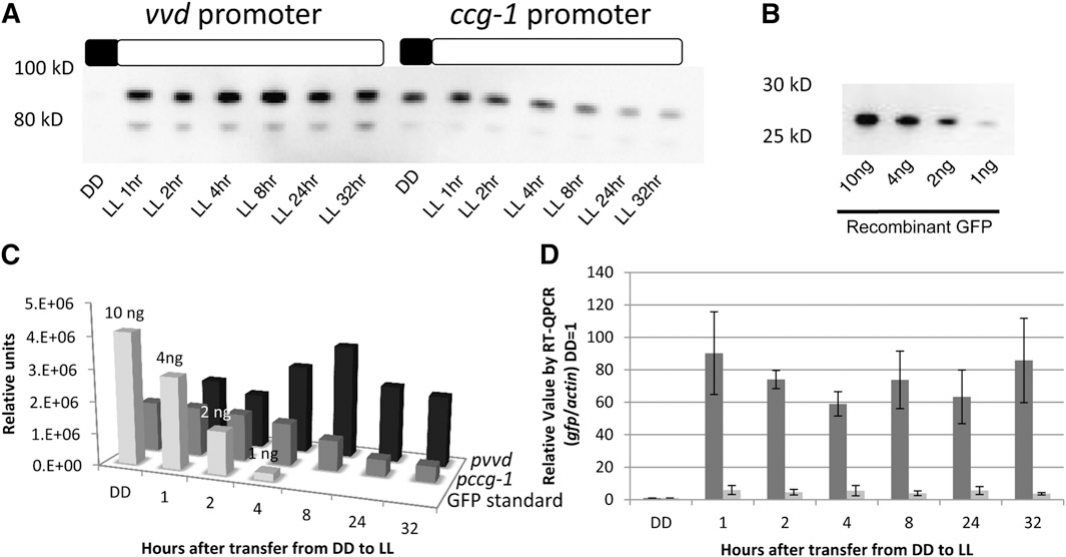
The *vvd* promoter drives stronger expression than the *ccg-1* promoter. (A) Expression of GH5-1-GFP^His6^ under the *vvd* and *ccg-1* promoters was determined by measuring GFP levels at high light (LL, 25 μeinsteins·m^2^·s). Boxes described the light regime; black is lights off and white is lights on. (B) Purified GFP run on the same the gel as a standard. (C) Protein levels of GFP/CBH-1 under the *ccg-1* promoter (dark gray) and GFP/CBH-1 under the *vvd* promoter (black) were compared with the standard GFP control (light gray) (Clontech) and quantitated using Image Lab Softwear (Bio-Rad). (D) Transcriptional levels were determined measuring induction of *gfp* under *vvd* (dark gray) and *ccg-1* (light gray) promoters using RT-qPCR analysis compared with actin (*n* = 3, error bars represent ± 1 SE). Boxes describe the light regime; black is lights off, and white is lights on. DD is dark conditions, LL is 25 μeinsteins·m^2^·s.

The light-induced and glucose-repressed *ccg-1* promoter (Loros *et al.* 1989; McNally and Free 1988; Sun *et al.* 2011) is widely used for overexpression and was recently used to drive expression of the endoglucanase GH5-1 (Sun *et al.* 2011). We compared *pvvd*-driven expression of *gh5-1* with that driven by induced and derepressed *pccg-1*; although we were not able to attain a complete repression of protein expression under the *ccg-1* promoter before induction, the level of transcription from the induced *vvd* promoter was 10-fold greater than from fully active *ccg-1* and 90-fold above background (Figure 3, A and D). Protein levels arising from the *vvd* promoter (0.4−0.9 ηg/μL) were on average 2- to 8-fold greater than from the *ccg-1* promoter (0.1−0.4 ηg/μL; Figure 3, A−C).

### Use of the vvd promoter

**Figure 4:**
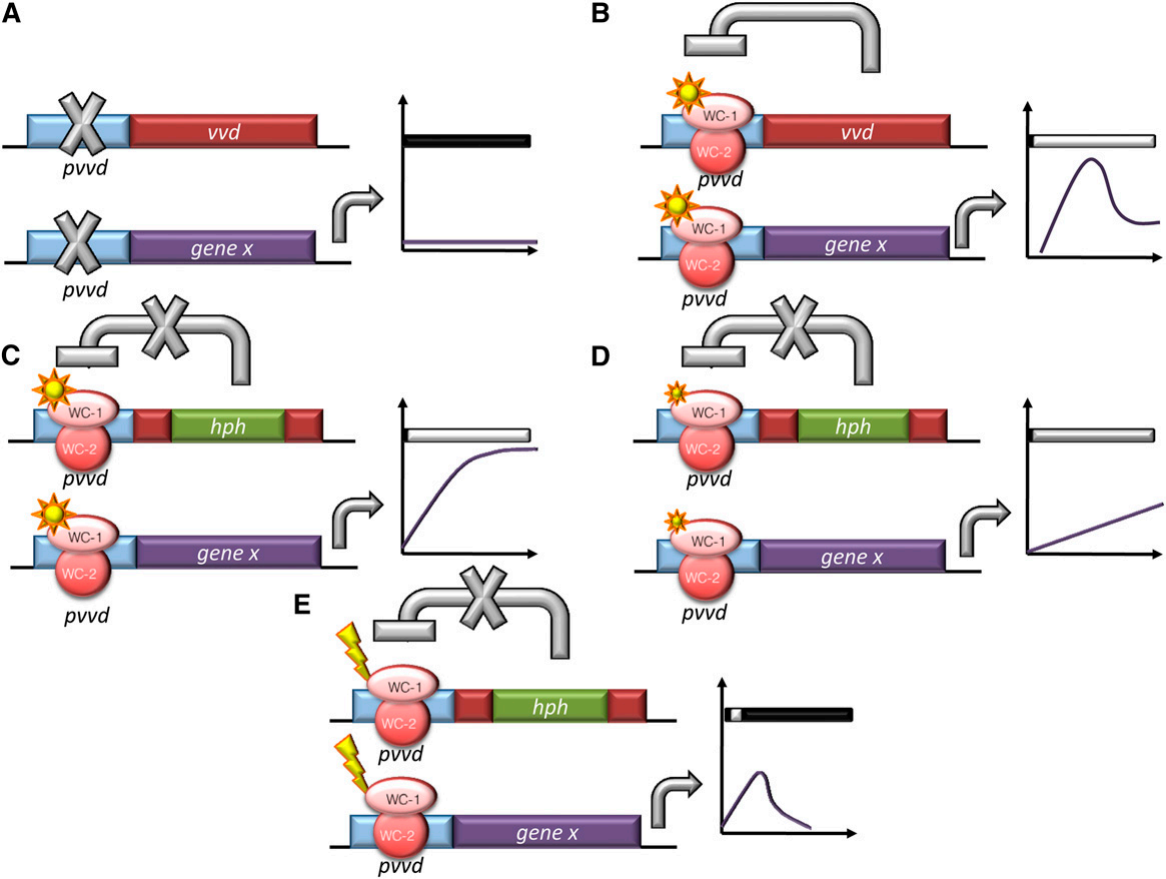
Outcomes of expression conditions from the *vvd* promoter in Neurospora. Schematic representation of the predicted as well as actual output from the *vvd* promoter in: (A) DD conditions; In DD, all genes driven by the *vvd* promoter are not induced. (B) LL conditions in the WT strain; the light activated WCC rapidly and strongly turns on expression at the *vvd* promoter. This leads to the production of VVD, which acts back on the WCC to inhibit the activation at the *vvd* promoter (photoadaptation). (C) LL conditions in the *Δvvd* strain; in the *vvd* KO strain, light activates the *vvd* promoter and because no VVD is produced, there is no photoadaptation and the gene of interest is continually expressed. (D) VLL conditions in the *Δvvd* strain; lower levels of light cause a lower level of expression, demonstrating that the system is regulatable by changing the level of light. (E) LP conditions in the *Δvvd* strain; after activation of the *vvd* promoter, the light is turned off and the promoter activity is repressed. Graphs represent levels of *gene x* (the gene of interest) under the *vvd* promoter in each condition described. DD is dark conditions, LL is 25 μeinsteins·m^2^·s, VLL is 0.01 μeinsteins·m^2^·s and LP is LL for 1 hr.

The light-inducible *vvd* promoter has a wide dynamic range that can be increased another two-fold when VVD’s autoregulation is eliminated in the *vvd* knockout strain (Figures 2 and 4, B and C). Expression of both exogenous and endogenous genes under the *vvd* promoter is indeed greater than that from *ccg*-1, even after 32 hr (Figure 3). Similarly, although *qa-2* is commonly used as a tunable promoter turned on and off with the addition or exclusion of quinic acid (Aronson *et al.* 1994; Shi *et al.* 2010), the *vvd* promoter is turned on more strongly and rapidly with light and is also repressed at a faster rate when returned to DD than is the *qa-2* promoter after inducer wash out [Figures 2 and 4, A and D and (Hong *et al.* 2008)]. Application and removal of the *vvd* inducer, light, is indeed tunable, rapid, and easy (Figure 4E). By these metrics, the *vvd* promoter compares favorably with existing regulated promoters currently available for use in Neurospora.

High levels of protein expression are useful for manufacturing antibiotics, insulin, and a host of academic pursuits. *pvvd*-driven expression of exogenous proteins could prove useful to those outside the fungal field as posttranslational modifications are maintained in Neurospora. Filamentous fungi can be engineered to secrete high levels of protein for a wide range of industrial applications (Arora 2003; Tian *et al.* 2009). Specifically, *N. crassa* has been shown to be useful for the expression and purification of a functional lignocellulolytic enzyme that can be used in the biofuel industry, where much research is aimed at using cellulosic materials to produce alcohol or other carbon-based fuels (Sun *et al.* 2011). As a tool in the field of filamentous fungi, we believe the use of the *vvd* promoter will be a useful instrument for those interested in efficient and regulatable gene expression.

## Supplementary Material

Supporting Information
