# Supplementary material for: Light-Inducible System for Tunable Protein Expression in Neurospora crassa
Source: G3 (Bethesda). 2012 Oct 1;2(10):1207–12. doi: 10.1534/g3.112.003939 (PMC3464113; doi:10.1534/g3.112.003939)
Supplement: Supporting Information [file supp_2_10_1207__index.html]

Supporting Information 

# Light-Inducible System for Tunable Protein Expression in *Neurospora crassa*

## Supporting Information for Hurley *et al.*, 2012

**Files in this Data Supplement:**

- File S1 - Supporting Results and Discussion, Figure S1, and Table S1 (PDF, 214 KB)
- Figure S1 - Constructs used to examine vvd promoter-driven gene expression (PDF, 136 KB)
- Table S1 - Vectors and Primers (PDF, 121 KB)
