# Supplementary material for: Light-Inducible System for Tunable Protein Expression in Neurospora crassa
Source: G3 (Bethesda). 2012 Oct 1;2(10):1207–12. doi: 10.1534/g3.112.003939 (PMC3464113; doi:10.1534/g3.112.003939)
Supplement: Supporting Information [file supp_2.10.1207_FigureS1.pdf]

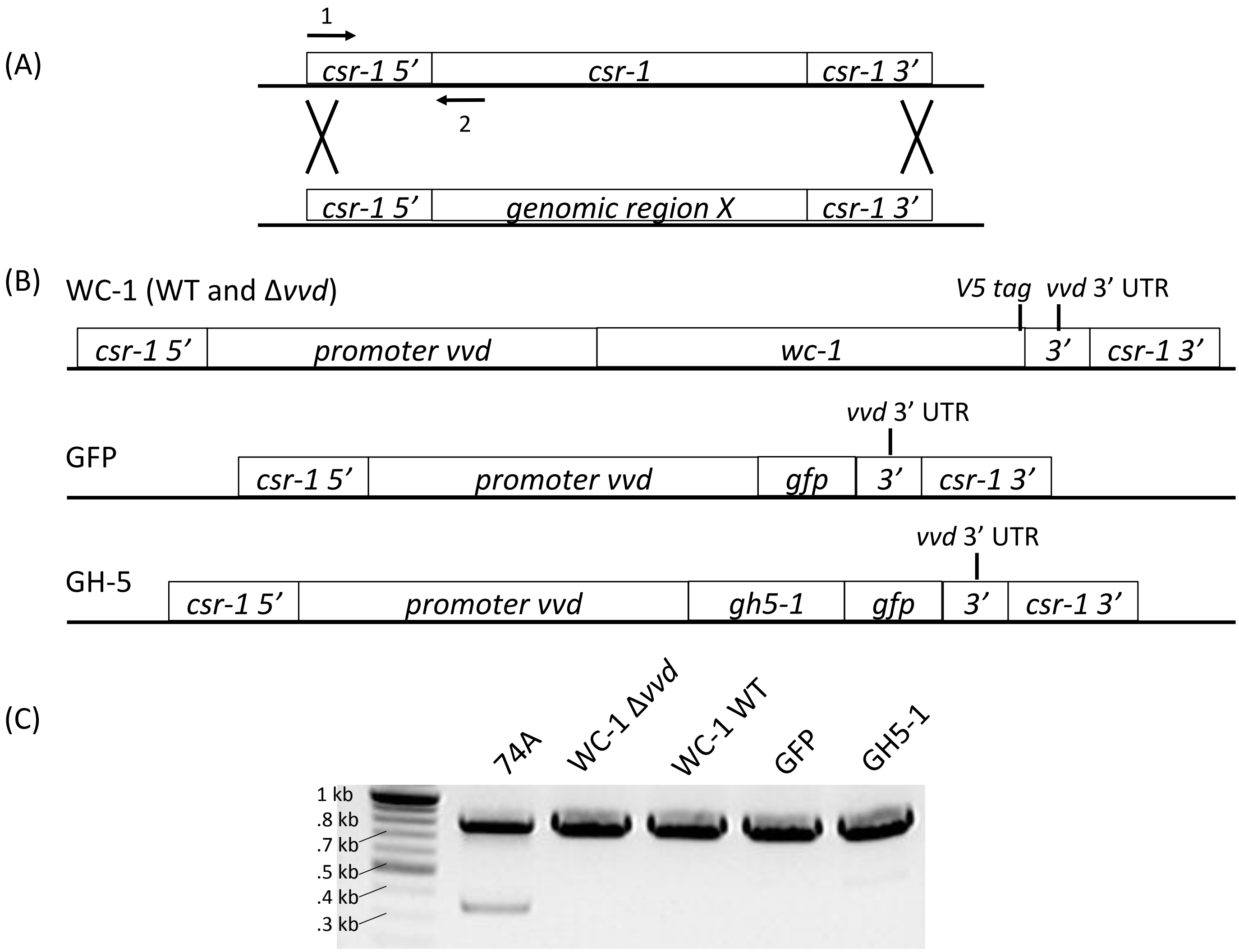

Figure S1 Constructs used to examine *vvd* promoter-driven gene expression. (**A**) Constructs bearing either *wc-1, gfp* or *gh5-1* were transformed into a WT or *Δvvd* strain at the *csr-1* locus (Bardiya and Shiu 2007). The arrows represent primers used in the PCR genotyping analysis in (**B**). Representation of constructs designed to test expression from the *vvd* promoter. Cassette containing *gh5-1* is based off of the design from Sun et. al. using the *vvd* promoter in place of the *ccg-1* promoter (Sun *et al.* 2011). (**C**) Genomic DNA samples from *wt*, *Δvvd wc-1*^{v5} *, wt wc-1*^{v5}*, gfp* and *gh5-1* strains were subjected to PCR analysis to examine the *csr-1* locus. Bands at 350 bp represent the presence of the *csr-1* locus while bands at 750 bp represent the presence of the *actin* locus (+ control).

Bardiya, N., and P. K. Shiu, 2007 Cyclosporin A-resistance based gene placement system for *Neurospora crassa*. Fungal Genet Biol **44:** 307-314.

Sun, J., C. M. Phillips, C. T. Anderson, W. T. Beeson, M. A. Marletta *et al.*, 2011 Expression and characterization of the *Neurospora crassa* endoglucanase GH5-1. Protein Expr Purif **75:** 147-154.
