# Supplementary material for: Light-Inducible System for Tunable Protein Expression in Neurospora crassa
Source: G3 (Bethesda). 2012 Oct 1;2(10):1207–12. doi: 10.1534/g3.112.003939 (PMC3464113; doi:10.1534/g3.112.003939)
Supplement: Supporting Information [file supp_2.10.1207_TableS1.pdf]

Table S1 Vectors and Primers

PVVD-GH-5:
TCGCGCGTTTCGGTGATGACGGTGAAAACCTCTGACACATGCAGCTCCCGGAGACGGTCACAGCTTGTCTG
TAAGCGGATGCCGGGAGCAGACAAGCCCGTCAGGGCGCGTCAGCGGGTGTTGGCGGGTGTCGGGGCTGGCT
TAACTATGCGGCATCAGAGCAGATTGTACTGAGAGTGCACCATACCACAGCTTTTCAATTCAATTCATCAT
TTTTTTTTTATTCTTTTTTTTGATTTCGGTTTCTTTGAAATTTTTTTGATTCGGTAATCTCCGAACAGAAG
GAAGAACGAAGGAAGGAGCACAGACTTAGATTGGTATATATACGCATATGTAGTGTTGAAGAAACATGAAA
TTGCCCAGTATTCTTAACCCAACTGCACAGAACAAAAACCTGCAGGAAACGAAGATAAATCATGTCGAAAG
CTACATATAAGGAACGTGCTGCTACTCATCCTAGTCCTGTTGCTGCCAAGCTATTTAATATCATGCACGAA
AAGCAAACAAACTTGTGTGCTTCATTGGATGTTCGTACCACCAAGGAATTACTGGAGTTAGTTGAAGCATT
AGGTCCCAAAATTTGTTTACTAAAAACACATGTGGATATCTTGACTGATTTTTCCATGGAGGGCACAGTTA
AGCCGCTAAAGGCATTATCCGCCAAGTACAATTTTTTACTCTTCGAAGACAGAAAATTTGCTGACATTGGT
AATACAGTCAAATTGCAGTACTCTGCGGGTGTATACAGAATAGCAGAATGGGCAGACATTACGAATGCACA
CGGTGTGGTGGGCCCAGGTATTGTTAGCGGTTTGAAGCAGGCGGCAGAAGAAGTAACAAAGGAACCTAGAG
GCCTTTTGATGTTAGCAGAATTGTCATGCAAGGGCTCCCTATCTACTGGAGAATATACTAAGGGTACTGTT
GACATTGCGAAGAGCGACAAAGATTTTGTTATCGGCTTTATTGCTCAAAGAGACATGGGTGGAAGAGATGA
AGGTTACGATTGGTTGATTATGACACCCGGTGTGGGTTTAGATGACAAGGGAGACGCATTGGGTCAACAGT
ATAGAACCGTGGATGATGTGGTCTCTACAGGATCTGACATTATTATTGTTGGAAGAGGACTATTTGCAAAG
GGAAGGGATGCTAAGGTAGAGGGTGAACGTTACAGAAAAGCAGGCTGGGAAGCATATTTGAGAAGATGCGG
CCAGCAAAACTAAAAAACTGTATTATAAGTAAATGCATGTATACTAAACTCACAAATTAGAGCTTCAATTT
AATTATATCAGTTATTACCCTATGCGGTGTGAAATACCGCACAGATGCGTAAGGAGAAAATACCGCATCAG
GAAATTGTAAACGTTAATATTTTGTTAAAATTCGCGTTAAATTTTTGTTAAATCAGCTCATTTTTTAACCA
ATAGGCCGAAATCGGCAAAATCCCTTATAAATCAAAAGAATAGACCGAGATAGGGTTGAGTGTTGTTCCAG
TTTGGAACAAGAGTCCACTATTAAAGAACGTGGACTCCAACGTCAAAGGGCGAAAAACCGTCTATCAGGGC
GATGGCCCACTACGTGAACCATCACCCTAATCAAGTTTTTTGGGGTCGAGGTGCCGTAAAGCACTAAATCG
GAACCCTAAAGGGAGCCCCCGATTTAGAGCTTGACGGGGAAAGCCGGCGAACGTGGCGAGAAAGGAAGGGA
AGAAAGCGAAAGGAGCGGGCGCTAGGGCGCTGGCAAGTGTAGCGGTCACGCTGCGCGTAACCACCACACCC
GCCGCGCTTAATGCGCCGCTACAGGGCGCGTCGCGCCATTCGCCATTCAGGCTGCGCAACTGTTGGGAAGG
GCGATCGGTGCGGGCCTCTTCGCTATTACGCCAGCTGGCGAAAGGGGGATGTGCTGCAAGGCGATTAAGTT
GGGTAACGCCAGGGTTTTCCCAGTCACGACGTTTCTGTGAAACGGCGGTAAATCATGGGCATGATTTCGTT
GAGTGTTTGGCTAAGAACAAGGAGGGAAACGCAAGTTAGCCGCGTTCGTATACAAGCGAGCAGTTCTCGAG
AACGTACTAGTTGAAGGTGCCATCGGCGATCTCTTGCATCAAAGTCGACGATGCACCCCAAGGTTCATTGT
TGGTGGCCTCGCGCACCTGGGTTCGCTGTCAGCTCCCGTTCCATGGTCTCTTGTCTGGGATCAAGTATATG
GCGAAGGGCGCAAGAGAATGGTGCCAGTGTGGCGCGTGGATACGGGCATACCTTGGACTCCATCTCGGTAT
AGTTCATGACAGCTGGTTGGAGCCATTGTCAGCACCCTGAACACGAAATCATGAGGGAGTCTTGGTTCTGG
ACTAGGTGGTCACCTACCATTTTGAACCTTTCGTACGCCAGCCTTGATGTCGTAGAGTGTCAGGTTGGACA
CGGTATCCTTGAGGCTATTGAAATCCATTGCGTAAACGTGTCTATCGCCGGAGTTGCTGGGAATTGTTCTG
ATGGGCTAAGGTAATTCTTCTCTAGATCCAATCTGAGCCTCGCGGTGATGATTGCCAGACGGGCGGGATGC
TGTGGCGACTTGGTGGTTTTTGTTCGGGCTGATCGCTTTCAGGGCGACGTTTCACGGTCTTGTCGGGATCA
GATGGACATTGAGGTGGCCTAGCGTCAGGGTGCCGCTGAGCGCTGACCTCAACTGACGCCGTCCAAGGCCG
ACCCCTGCGCTTGACCCAACACCCTGACACCAAGAAGCCAGTGGAACCCAGGAGGTCTGCCAAGACTTTGA
AAGCTTGGCACGAGCGTCTCAGCTCTTTGAAGCCTGAGTTTTCAGGCAAGAATTCTGCTTGTAATTGCCGG
GCCGGCCGACAACGCGGGGCGGCGGGAGGGGACGCTCGGCCGCCGGAGCTCGCTTCCATGCATTTTGGATG
GAGTAAAAATTGTTAAACACCCGACCCGACTCCGTTCCAGTGGCATCAAACACAAGCCAGTGGCATCAAAC
ACAAGCCGTCTTTAACATCGGAGTATACTACACACACACGCACTCTCAGCAGTGTGTTTTTGGAAGATCGA
ATGCTGACTTAACACAGGCAACCTCTTCACGAAGACCCGGCTTGCCGTTTATTATTCGGTGTAACCAAGCG
CTGGGACTGTGCACCGTGTCACATCGAGATTTCAAACTGCTCTGCGGTCGTGCAGTCACCGGTTATCATGG
AACTAATTGGAAACCAAGCGGCAGGTTGAGCAAAGTCTTGCCCTGAAAGTTGGCACATTAGAGCCATCCCG
TTTGCGGTTGCAGATGGATGAAAAACGAGCGGAATACACGGGAACAGGGCAAGGTGACCATGACATGGGCG
AAAGGTAAGTAGCCGGGGACAGACGCAACCCGCGCACATCCCGAAAGGGGAAGTTGCGATAATCCCGTGTT
GTGACTTCTCCCTCTTCCCTCGATGATGTGCCGACGCCAACGCCATCACTTATTCACGCCAGCACTGTGAT
TCGAGCTTGCCTGCTTCCTGGCGTCCTATGGCCTCCCGTATCCTGCTGGTGATCATCCCAATCTGTCAAGT
CTCATTTCCCCTCATTCCCATCACGATGAGGAGCCTGAGACTTTTCGGGAAAAGAGGGGGCGTCGATAGTT
TGGTCCGTTTTGTCGACGGTATTCATGTGATGTCCCTACAGTTGCATGAGACCAGGCCGCCCTGTTGTTGT
TCATGGTTCTCTCCCAGTCCCAAGGGAGCCAGGCTGGGGCGCCCCCCTCCCCCCCTCCTTCTTTTGTCCTT
CCACAACTTCATGACCCCCCCATGCGCCTCTGCCTAAAGGTCTACCACCTACCGGACCCTCTGCTCATCAA
GACCCCCAAGATGCCCCGCCCGCTTTATCGCCATACAGTAAATTGACTGTACATATACCCGACGATAACCT

GGTTGCTGTTGGATCCAAACTCGGGCTGTCGAGCATACAAGCGAGAAGCGTGGGTCACCTCACGGCTGGTC
GGCGCAAAGGGACGAGGTAGGTTTGTAGTGTAGCAAGGGGGTTCGGCCTTGACGCCTACAAGGGAGCAGAT
GGAATGGGAACACCATAACAACCGAGCCCAGAACCAAGGCATCACCACATGTCTTTAGCGCAACGATATCA
GCAAGCTCTCCGAACACATATGAAGATGGGTTCCGCTATTATGGTCCTGGCGGTGTTGAGTACTTGAGGTA
CATATCTACCTACCTACCTACCTACCTACCTACCTACCTACCTACCTACCTACCTACCAACCAACCAACCT
CCCCACCAACCTCTCGGGTCGGGCAGATTAACGTTGGTGGTTAAGGTGTCAAGTGTCGTATGTCCGGTGGT
GGTGGTGGTGGTGGTGGTGCGCGGTGCGCATTTGACAGCCGGTGACGGGACAACTTGAAAGGAACCGAAAC
CGAAGAGACAACTTGACAGATGGAAGTGACTAAGTAACTCCCTAGAGCACTAGGGTTGACGTTGCAGTAAA
AACGTGCCCGTTTTCTTTCCCTATCTGCACTGTCCGCTGGCTGGTTGGTGCGGTTTCAATTGTTCGTGCTG
GCGCGTGGGTCCCGTTCTTGGTTCGATCCACGGCGCTGGTGCGATCGCTGCGGATCTGGGTCCCTCGATGG
TTTAGCAGCTGCGATCGGTCAGCATCGCTGTCCCCATTCTACACTGCCATCCAAAATCAGAAAGTGTCCCC
CAAGTTCGCTTGGGGGTTCCATTGGTCTTGGCGGCATCCAGATTTGCCTGCATTCTCACGCCGTCTGCTGC
TCCTTCTTCTCCTATTTGTCTCCGTGCTGTCCGCGAATGGGTACGTTTTTCAACGTTTTGGCCTCTTGGCC
ATGACCCTCTTCGGTTTCTGGATCGAACTTTGTTCGGTTATTAAGTACCTAGGTACCTACGTCTATGACTC
CATCGGCCTCTCTTTCCTCCCTTCGGACTTTTGCATATCCACAATTGGCAACTCTCACCGATCTCCATCTC
GTCAACCTCCTCTCGTGCTTTGATAACGCTTATACTATCTTTCTCTTCTCTGTTAGCATTCATTTGAAGAC
ACTATCTTAATAAGCAAAAACCCTAAGACGCCATCGATCTGAACAATAAAAGTCACCACGGAATTGTTCAC
CAAAAGTGTGAAGCATATATGGCATCATCAGCCCATTGAGAGACTACTATGTAATCAAGATCAAGACTCTT
CTTCCATGACTTTTGGTGCCATTGGTCTTGGGTTGGGGGTGTAATGGTCAAATAAACGGCAAGGGGGTGGT
CAGCCCTGAGCGTCGATGAAGAGCGTGATGATAAAAACCTATACGGTGCAAGTTGTCGATAAAGACGGGGC
ACATGATGGGGATTCTTGGATGCCAGGGTCTTCCACCTAGTCCAGGGTGTCAAAGGAAATGCGGTCCCATT
ACTGGATCCATCCGAACTGCTCGAACATCGGGCCACTCCCGTTGTGTCCTCTGAGTTTCCCGATCTCAAGA
GCTTCCCGATGCAGGCTGTGATCACAGATCCTGGCCTCGGCGCTGAGACGGGATGATGGATACCGCTGTAC
ACTAGATACGGGACAGCAGAGCAAGCGGCACCCGGACAATGGAACAGCAGCAAGCAGTCACTCTTCAACAA
AAAAAAAAATCAAAAAATCGAAACGGCCATGTCGCCGGCATACCACCCACAGCCTGGCTCCCGCGTTTTGT
CCATGGCTCACACAAAGACCTCCCATCTCTTGTCCCAGCCGTGGTCTCTTTGTGTACCCTCAGGCCACCCT
TGCTACCTACGTACATGGAACGTGTAAAGGCGTCGTGTACCGTTCGATTGGATCGGCGTCTTGTCTCTGGC
AACTGTCTCTCTTTTTTTCTGTATGTCCCTGGCCCCCCGTTGGCCTGCCCTGCCTGGCTCTTGAGCTATGG
TGTCTCTCCATACAGGACCCTGGGCATCCCAAACACCCCTGGCTCCCACTTCAAAATGAAGGCTACGATTC
TTGCCAGCACCTTCGCCGCTGGTGCCCTCGCCCAGAGCGGTGCCTGGGGCCAATGCGGCGGTAACGGTTGG
TCCGGCGCTACCAGCTGCATCTCCGGATACGCCTGCAACTACGTGAACGATTGGTATAGCCAGTGCCAGCC
TGGTACTGCCGCTCCTACCACCACTGCCGCCGCCACCACCCTCGTCACCTCGACCAAGACCGCCCCTCCTG
CTAGCACCACCACTGCCACCGCCTCCGGCAAGTTCAAGTGGTTCGGTGTCAACGAGGCCGGCGGTGAGTTT
GGTGATGGTATCTTCCCCGGAAGATGGGGCACTGAGTTCACATTCCCTGACACCAACACCATCCAGACTCT
CCGCAGCCAGGGTTACAACATCTTCCGTGTTGGCTTCGCCATGGAGCGCCTTGTCCCCAACACCCTGACGT
CGTCTTTCGATAACGGCTATCTCACCAACCTCACCCAGGTTGTCAACTCTGTTACCAACTCTGGTGCCTAT
ATTGTTCTCGATCCCCACAACTATGGCCGTTACTATGGCAAAATCATCACCGATACCGATGCCTTCAAGAC
TTTCTGGCAAAATGTTGCTGCCAAGTTTGCTTCCAACTCCAAGGTCATCTTCGATACCAACAACGAGTACA
ACACCATGGACCAGACCCTTGTCCTCAACCTCAACCAGGCCGCCATTGACGGTATTCGTGCGGCCGGCGCC
ACTTCGCAGTACATTTTCGTCGAGGGTAACCAATGGACTGGCGCCTGGTCATGGAATGTGACCAACACCAA
TTTGGCTGCCCTTACCGATCCCGAGAACAAGATCGTCTACGAGATGCACCAGTACCTCGACTCCGATAGCT
CCGGTACCAGCACTGCCTGCGTTTCCTCCGAGATCGGTGTTCAGCGCATTGTTGGTGCCACTGCCTGGCTC
CGCGCCAATGGCAAAAAGGGTGTCCTCGGCGAGTTCGCTGGTGGTGCCAACTCAGTCTGCAAGGCTGCCGT
TACTGGCCTTCTTGAGCACCTCAAGGCTAACACTGACGTCTGGGAGGGTGCCCTTTGGTGGGCTGCCGGTC
CCTGGTGGGGTGACTACATGTATAGCTTTGAGCCTCCTTCGGGCACTGGCTATACCTACTACAACAGCCTT
CTCAAGACCTATACCCCTGGATCCGTGAGCAAGGGCGAGGAGCTGTTCACCGGGGTGGTGCCCATCCTGGT
CGAGCTGGACGGCGACGTAAACGGCCACAAGTTCAGCGTGCGCGGCGAGGGCGAGGGCGATGCCACCAACG
GCAAGCTGACCCTGAAGTTCATCTGCACCACCGGCAAGCTGCCCGTGCCCTGGCCCACCCTCGTGACCACC
CTGACCTACGGCGTGCAGTGCTTCAGCCGCTACCCCGACCACATGAAGCAGCACGACTTCTTCAAGTCCGC
CATGCCCGAAGGCTACGTCCAGGAGCGCACCATCTTCTTCAAGGACGACGGCACCTACAAGACCCGCGCCG
AGGTGAAGTTCGAGGGCGACACCCTGGTGAACCGCATCGAGCTGAAGGGCATCGACTTCAAGGAGGACGGC
AACATCCTGGGGCACAAGCTGGAGTACAACTTCAACAGCCACAACGTCTATATCATGGCCGACAAGCAGAA
GAACGGCATCAAGGTGAACTTCAAGATCCGCCACAACGTCGAGGACGGCAGCGTGCAGCTCGCCGACCACT
ACCAGCAGAACACCCCCATCGGCGACGGCCCCGTGCTGCTGCCCGACAACCACTACCTGAGCACCCAGTCC
GTCCTGAGCAAAGACCCCAACGAGAAGCGCGATCACATGGTCCTGCTGGAGTTCGTGACCGCCGCCGGGAT
CACTCTCGGCATGGACGAGCTGTACAAGCATCATCACCATCACCATTAATTAATTAACACCCACCCTTCTT
CACTGCATCGTCATCACTGTCGCTTTGATGGTTGATGTGATTACGCAAACTGATGGTTGATGAGATCACGT
AAACTGATGTCTTTTCCTAGACAGTTTGCAGGTCATGTGAATGTTATCGCCAACACATTGAATGCTAAACA

GATTACCTCCAACGCCTAGATGAAACCAAATTACCCTTGCCTTCTTTTTTTTTACGCCGTTAATGCAGTTG
TGATAGAGACCATGCAACGTCTTCTAAACGGAGACACAAACACATTCGAGTCGATCAGATCGAAATGACTT
GTTCCTGTACCTCGTCACCATCCAGCCACCGTCTGAAATAGACGCAGTTGCTGGGGCTGACAGGTCCGCCT
TCCTCGCACATATCATAGACAGGGCACCGCCCACAAGGCGCCTCCATAAGGCCACTTGCACTCGGCTCAAC
TCCCATAAATGAGGTAACAATGCCTCCTTTGGTTGGATCATGGTCGAGGCGTCGTTGATTGCACGTCCTCG
GATCCTGTTTGGGGATGCGCGTCACACGGTACCCCTTGCGCCCATTGACCTTGACAGGCTCTACTAAGCCA
TCGTAGTATAGCACATCCACCAACTGCTGGATATCCGCCTCGCTCAACGTCGTATTATTCGTGATGCCGGC
GTGGTGGATGAACTTTGCAATGTCCGACACAGTCGGGTACGACTTGTAACCGGCCGGGAGAGGCAGCAATT
GCGGCTTGCCGGGCCTGGTCTTCTTGGCCGGTGCCGGTGCCGCCGTGTCGTCGTTGGAAATCTCGCCCGCG
CTGCGCTTCGTCCCCTTGGAAATATCCTCCGAGTGGGGAATGGTCCCCTGAACCACGCCCTTCTTGGGATT
CTTTTCACGCTGCTGTGCTCCTCCTCCTTGTGCTCCTACTCCTGGTACTCCAGCACCGCCGCCGTGGGAAG
TATATGTCGATTTCTGCTTGATAAAGTCAAACACAATTTCCTGTAGCTCGTTGATGAAGGCCGTTGTAAAA
CGACGGCCAGTGAGCGCGCGTAATACGACTCACTATAGGGCGAATTGGGTACCGGGCCCCCCCTCGAGGTC
GACGGTATCGATAAGCTTGATATCGAATTCCTGCAGCCCGGGGGATCCACTAGTTCTAGAGCGGCCGCCAC
CGCGGTGGAGCTCCAGCTTTTGTTCCCTTTAGTGAGGGTTAATTGCGCGCTTGGCGTAATCATGGTCATAG
CTGTTTCCTGTGTGAAATTGTTATCCGCTCACAATTCCACACAACATAGGAGCCGGAAGCATAAAGTGTAA
AGCCTGGGGTGCCTAATGAGTGAGGTAACTCACATTAATTGCGTTGCGCTCACTGCCCGCTTTCCAGTCGG
GAAACCTGTCGTGCCAGCTGCATTAATGAATCGGCCAACGCGCGGGGAGAGGCGGTTTGCGTATTGGGCGC
TCTTCCGCTTCCTCGCTCACTGACTCGCTGCGCTCGGTCGTTCGGCTGCGGCGAGCGGTATCAGCTCACTC
AAAGGCGGTAATACGGTTATCCACAGAATCAGGGGATAACGCAGGAAAGAACATGTGAGCAAAAGGCCAGC
AAAAGGCCAGGAACCGTAAAAAGGCCGCGTTGCTGGCGTTTTTCCATAGGCTCCGCCCCCCTGACGAGCAT
CACAAAAATCGACGCTCAAGTCAGAGGTGGCGAAACCCGACAGGACTATAAAGATACCAGGCGTTTCCCCC
TGGAAGCTCCCTCGTGCGCTCTCCTGTTCCGACCCTGCCGCTTACCGGATACCTGTCCGCCTTTCTCCCTT
CGGGAAGCGTGGCGCTTTCTCATAGCTCACGCTGTAGGTATCTCAGTTCGGTGTAGGTCGTTCGCTCCAAG
CTGGGCTGTGTGCACGAACCCCCCGTTCAGCCCGACCGCTGCGCCTTATCCGGTAACTATCGTCTTGAGTC
CAACCCGGTAAGACACGACTTATCGCCACTGGCAGCAGCCACTGGTAACAGGATTAGCAGAGCGAGGTATG
TAGGCGGTGCTACAGAGTTCTTGAAGTGGTGGCCTAACTACGGCTACACTAGAAGGACAGTATTTGGTATC
TGCGCTCTGCTGAAGCCAGTTACCTTCGGAAAAAGAGTTGGTAGCTCTTGATCCGGCAAACAAACCACCGC
TGGTAGCGGTGGTTTTTTTGTTTGCAAGCAGCAGATTACGCGCAGAAAAAAAGGATCTCAAGAAGATCCTT
TGATCTTTTCTACGGGGTCTGACGCTCAGTGGAACGAAAACTCACGTTAAGGGATTTTGGTCATGAGATTA
TCAAAAAGGATCTTCACCTAGATCCTTTTAAATTAAAAATGAAGTTTTAAATCAATCTAAAGTATATATGA
GTAAACTTGGTCTGACAGTTACCAATGCTTAATCAGTGAGGCACCTATCTCAGCGATCTGTCTATTTCGTT
CATCCATAGTTGCCTGACTCCCCGTCGTGTAGATAACTACGATACGGGAGGGCTTACCATCTGGCCCCAGT
GCTGCAATGATACCGCGAGACCCACGCTCACCGGCTCCAGATTTATCAGCAATAAACCAGCCAGCCGGAAG
GGCCGAGCGCAGAAGTGGTCCTGCAACTTTATCCGCCTCCATCCAGTCTATTAATTGTTGCCGGGAAGCTA
GAGTAAGTAGTTCGCCAGTTAATAGTTTGCGCAACGTTGTTGCCATTGCTACAGGCATCGTGGTGTCACGC
TCGTCGTTTGGTATGGCTTCATTCAGCTCCGGTTCCCAACGATCAAGGCGAGTTACATGATCCCCCATGTT
GTGCAAAAAAGCGGTTAGCTCCTTCGGTCCTCCGATCGTTGTCAGAAGTAAGTTGGCCGCAGTGTTATCAC
TCATGGTTATGGCAGCACTGCATAATTCTCTTACTGTCATGCCATCCGTAAGATGCTTTTCTGTGACTGGT
GAGTACTCAACCAAGTCATTCTGAGAATAGTGTATGCGGCGACCGAGTTGCTCTTGCCCGGCGTCAATACG
GGATAATACCGCGCCACATAGCAGAACTTTAAAAGTGCTCATCATTGGAAAACGTTCTTCGGGGCGAAAAC
TCTCAAGGATCTTACCGCTGTTGAGATCCAGTTCGATGTAACCCACTCGTGCACCCAACTGATCTTCAGCA
TCTTTTACTTTCACCAGCGTTTCTGGGTGAGCAAAAACAGGAAGGCAAAATGCCGCAAAAAAGGGAATAAG
GGCGACACGGAAATGTTGAATACTCATACTCTTCCTTTTTCAATATTATTGAAGCATTTATCAGGGTTATT
GTCTCATGAGCGGATACATATTTGAATGTATTTAGAAAAATAAACAAATAGGGGTTCCGCGCACATTTCCC
CGAAAAGTGCCACCTGAACGAAGCATCTGTGCTTCATTTTGTAGAACAAAAATGCAACGCGAGAGCGCTAA
TTTTTCAAACAAAGAATCTGAGCTGCATTTTTACAGAACAGAAATGCAACGCGAAAGCGCTATTTTACCAA
CGAAGAATCTGTGCTTCATTTTTGTAAAACAAAAATGCAACGCGAGAGCGCTAATTTTTCAAACAAAGAAT
CTGAGCTGCATTTTTACAGAACAGAAATGCAACGCGAGAGCGCTATTTTACCAACAAAGAATCTATACTTC
TTTTTTGTTCTACAAAAATGCATCCCGAGAGCGCTATTTTTCTAACAAAGCATCTTAGATTACTTTTTTTC
TCCTTTGTGCGCTCTATAATGCAGTCTCTTGATAACTTTTTGCACTGTAGGTCCGTTAAGGTTAGAAGAAG
GCTACTTTGGTGTCTATTTTCTCTTCCATAAAAAAAGCCTGACTCCACTTCCCGCGTTTACTGATTACTAG
CGAAGCTGCGGGTGCATTTTTTCAAGATAAAGGCATCCCCGATTATATTCTATACCGATGTGGATTGCGCA
TACTTTGTGAACAGAAAGTGATAGCGTTGATGATTCTTCATTGGTCAGAAAATTATGAACGGTTTCTTCTA
TTTTGTCTCTATATACTACGTATAGGAAATGTTTACATTTTCGTATTGTTTTCGATTCACTCTATGAATAG
TTCTTACTACAATTTTTTTGTCTAAAGAGTAATACTAGAGATAAACATAAAAAATGTAGAGGTCGAGTTTA
GATGCAAGTTCAAGGAGCGAAAGGTGGATGGGTAGGTTATATAGGGATATAGCACAGAGATATATAGCAAA
GAGATACTTTTGAGCAATGTTTGTGGAAGCGGTATTCGCAATATTTTAGTAGCTCGTTACAGTCCGGTGCG

TTTTTGGTTTTTTGAAAGTGCGTCTTCAGAGCGCTTTTGGTTTTCAAAAGCGCTCTGAAGTTCCTATACTT
TCTAGAGAATAGGAACTTCGGAATAGGAACTTCAAAGCGTTTCCGAAAACGAGCGCTTCCGAAAATGCAAC
GCGAGCTGCGCACATACAGCTCACTGTTCACGTCGCACCTATATCTGCGTGTTGCCTGTATATATATATATAC
ATGAGAAGAACGGCATAGTGCGTGTTTATGCTTAAATGCGTACTTATATGCGTCTATTTATGTAGGATGAA
AGGTAGTCTAGTACCTCCTGTGATATTATCCCATTCCATGCGGGGTATCGTATGCTTCCTTCAGCACTACC
CTTTAGCTGTTCTATATGCTGCCACTCCTCAATTGGATTAGTCTCATCCTTCAATGCTATCATTTCCTTTG
ATATTGGATCATACTAAGAAACCATTATTATCATGACATTAACCTATAAAAATAGGCGTATCACGAGGCCC
TTTCGTC

PVVD-WC-1:
TCGCGCGTTTCGGTGATGACGGTGAAAACCTCTGACACATGCAGCTCCCGGAGACGGTCACAGCTTGTCTG
TAAGCGGATGCCGGGAGCAGACAAGCCCGTCAGGGCGCGTCAGCGGGTGTTGGCGGGTGTCGGGGCTGGCT
TAACTATGCGGCATCAGAGCAGATTGTACTGAGAGTGCACCATACCACAGCTTTTCAATTCAATTCATCAT
TTTTTTTTTATTCTTTTTTTTGATTTCGGTTTCTTTGAAATTTTTTTGATTCGGTAATCTCCGAACAGAAG
GAAGAACGAAGGAAGGAGCACAGACTTAGATTGGTATATATACGCATATGTAGTGTTGAAGAAACATGAAA
TTGCCCAGTATTCTTAACCCAACTGCACAGAACAAAAACCTGCAGGAAACGAAGATAAATCATGTCGAAAG
CTACATATAAGGAACGTGCTGCTACTCATCCTAGTCCTGTTGCTGCCAAGCTATTTAATATCATGCACGAA
AAGCAAACAAACTTGTGTGCTTCATTGGATGTTCGTACCACCAAGGAATTACTGGAGTTAGTTGAAGCATT
AGGTCCCAAAATTTGTTTACTAAAAACACATGTGGATATCTTGACTGATTTTTCCATGGAGGGCACAGTTA
AGCCGCTAAAGGCATTATCCGCCAAGTACAATTTTTTACTCTTCGAAGACAGAAAATTTGCTGACATTGGT
AATACAGTCAAATTGCAGTACTCTGCGGGTGTATACAGAATAGCAGAATGGGCAGACATTACGAATGCACA
CGGTGTGGTGGGCCCAGGTATTGTTAGCGGTTTGAAGCAGGCGGCAGAAGAAGTAACAAAGGAACCTAGAG
GCCTTTTGATGTTAGCAGAATTGTCATGCAAGGGCTCCCTATCTACTGGAGAATATACTAAGGGTACTGTT
GACATTGCGAAGAGCGACAAAGATTTTGTTATCGGCTTTATTGCTCAAAGAGACATGGGTGGAAGAGATGA
AGGTTACGATTGGTTGATTATGACACCCGGTGTGGGTTTAGATGACAAGGGAGACGCATTGGGTCAACAGT
ATAGAACCGTGGATGATGTGGTCTCTACAGGATCTGACATTATTATTGTTGGAAGAGGACTATTTGCAAAG
GGAAGGGATGCTAAGGTAGAGGGTGAACGTTACAGAAAAGCAGGCTGGGAAGCATATTTGAGAAGATGCGG
CCAGCAAAACTAAAAAACTGTATTATAAGTAAATGCATGTATACTAAACTCACAAATTAGAGCTTCAATTT
AATTATATCAGTTATTACCCTATGCGGTGTGAAATACCGCACAGATGCGTAAGGAGAAAATACCGCATCAG
GAAATTGTAAACGTTAATATTTTGTTAAAATTCGCGTTAAATTTTTGTTAAATCAGCTCATTTTTTAACCA
ATAGGCCGAAATCGGCAAAATCCCTTATAAATCAAAAGAATAGACCGAGATAGGGTTGAGTGTTGTTCCAG
TTTGGAACAAGAGTCCACTATTAAAGAACGTGGACTCCAACGTCAAAGGGCGAAAAACCGTCTATCAGGGC
GATGGCCCACTACGTGAACCATCACCCTAATCAAGTTTTTTGGGGTCGAGGTGCCGTAAAGCACTAAATCG
GAACCCTAAAGGGAGCCCCCGATTTAGAGCTTGACGGGGAAAGCCGGCGAACGTGGCGAGAAAGGAAGGGA
AGAAAGCGAAAGGAGCGGGCGCTAGGGCGCTGGCAAGTGTAGCGGTCACGCTGCGCGTAACCACCACACCC
GCCGCGCTTAATGCGCCGCTACAGGGCGCGTCGCGCCATTCGCCATTCAGGCTGCGCAACTGTTGGGAAGG
GCGATCGGTGCGGGCCTCTTCGCTATTACGCCAGCTGGCGAAAGGGGGATGTGCTGCAAGGCGATTAAGTT
GGGTAACGCCAGGGTTTTCCCAGTCACGACGATTTAAATTAGATCCAATCTGAGCCTCGCGGTGATGATTG
CCAGACGGGCGGATGCTGTGGCGACTTGGTGGTTTTTGTTCGGGCTGATCGCTTTCAGGGCGACGTTTCA
CGGTCTTGTCGGGATCAGATGGACATTGAGGTGGCCTAGCGTCAGGGTGCCGCTGAGCGCTGACCTCAACT
GACGCCGTCCAAGGCCGACCCCTGCGCTTGACCCAACACCCTGACACCAAGAAGCCAGTGGAACCCAGGAG
GTCTGCCAAGACTTTGAAAGCTTGGCACGAGCGTCTCAGCTCTTTGAAGCCTGAGTTTTCAGGCAAGAATT
CTGCTTGTAATTGCCGGGCCGGCCGACAACGCGGGGCGGCGGGAGGGGACGCTCGGCCGCCGGAGCTCGCT
TCCATGCATTTTGGATGGAGTAAAAATTGTTAAACACCCGACCCGACTCCGTTCTCCCTCTCGTCTCAGGT
CTTTGTTGTGCATCCATCGCCGTGTAGCAGATTTCCATCGTTCGTTTGTTCGGACTTTACCTTGTACTTTT
AGCTTCATTATCATCCTACAATCATCCAGGATTTCATCTTCCACAACCGGTACGTTATCATTTTTACTTTT
GTTGCCATGTTCTTCTTGAGCCTTTTCTGTCTAGAGGAGCTCTCCCTGCCGGCTGGGAGCTTGCCCAAAGC
TCCAAGCTCTATCTGTGGCAGAGCTCTGAGAGCTTCCATTCCCGGGATTTCTTGGCATCAGATTCAATTCG
AATGTCCTTTTTTCCATCCACGCTCGAGATGAGGGGTGAGGTTTGTTAGTTTGACAATACCACCACCGCCT
TTTGGTCCCTACCCCAGACATGGCCATCAAGGTCGTTAATGCCGTCCATCCCTCTTGTTCGGCTTGGGGTA
TCGTCCATGGCTCCGCAATGTCGTCAATGGCTGCATTGACAGAGGGGGACTCCAAATGAAAGAGGCAATCG
CAACGCTATGAAGACCCAACAAGCCATATGATGGAGCCATGGGTCTACGGTCCTTTGGCCCGGATGTCTTT
GGATAGCTCTTGCCAGCGCTTTAGCTGCCAACTATACACACACCGGAATTATACGATTTAGGTGACTGCAG
GATTAACCCTCACTAAAGGGACAGTGGCATCAAACACAAGCCAGTGGCATCAAACACAAGCCGTCTTTAAC
ATCGGAGTATACTACACACACACGCACTCTCAGCAGTGTGTTTTTGGAAGATCGAATGCTGACTTAACACA
GGCAACCTCTTCACGAAGACCCGGCTTGCCGTTTATTATTCGGTGTAACCAAGCGCTGGGACTGTGCACCG
TGTCACATCGAGATTTCAAACTGCTCTGCGGTCGTGCAGTCACCGGTTATCATGGAACTAATTGGAAACCA
AGCGGCAGGTTGAGCAAAGTCTTGCCCTGAAAGTTGGCACATTAGAGCCATCCCGTTTGCGGTTGCAGATG

GATGAAAAACGAGCGGAATACACGGGAACAGGGCAAGGTGACCATGACATGGGCGAAAGGTAAGTAGCCGG
GGACAGACGCAACCCGCGCACATCCCGAAAGGGGAAGTTGCGATAATCCCGTGTTGTGACTTCTCCCTCTT
CCCTCGATGATGTGCCGACGCCAACGCCATCACTTATTCACGCCAGCACTGTGATTCGAGCTTGCCTGCTT
CCTGGCGTCCTATGGCCTCCCGTATCCTGCTGGTGATCATCCCAATCTGTCAAGTCTCATTTCCCCTCATT
CCCATCACGATGAGGAGCCTGAGACTTTTCGGGAAAAGAGGGGGCGTCGATAGTTTGGTCCGTTTTGTCGA
CGGTATTCATGTGATGTCCCTACAGTTGCATGAGACCAGGCCGCCCTGTTGTTGTTCATGGTTCTCTCCCA
GTCCCAAGGGAGCCAGGCTGGGGCGCCCCCCTCCCCCCCTCCTTCTTTTGTCCTTCCACAACTTCATGACC
CCCCCATGCGCCTCTGCCTAAAGGTCTACCACCTACCGGACCCTCTGCTCATCAAGACCCCCAAGATGCCC
CGCCCGCTTTATCGCCATACAGTAAATTGACTGTACATATACCCGACGATAACCTGGTTGCTGTTGGATCC
AAACTCGGGCTGTCGAGCATACAAGCGAGAAGCGTGGGTCACCTCACGGCTGGTCGGCGCAAAGGGACGAG
GTAGGTTTGTAGTGTAGCAAGGGGGTTCGGCCTTGACGCCTACAAGGGAGCAGATGGAATGGGAACACCAT
AACAACCGAGCCCAGAACCAAGGCATCACCACATGTCTTTAGCGCAACGATATCAGCAAGCTCTCCGAACA
CATATGAAGATGGGTTCCGCTATTATGGTCCTGGCGGTGTTGAGTACTTGAGGTACATATCTACCTACCTA
CCTACCTACCTACCTACCTACCTACCTACCTACCTACCTACCAACCAACCAACCTCCCCACCAACCTCTCG
GGTCGGGCAGATTAACGTTGGTGGTTAAGGTGTCAAGTGTCGTATGTCCGGTGGTGGTGGTGGTGGTGGTG
GTGCGCGGTGCGCATTTGACAGCCGGTGACGGGACAACTTGAAAGGAACCGAAACCGAAGAGACAACTTGA
CAGATGGAAGTGACTAAGTAACTCCCTAGAGCACTAGGGTTGACGTTGCAGTAAAAACGTGCCCGTTTTCT
TTCCCTATCTGCACTGTCCGCTGGCTGGTTGGTGCGGTTTCAATTGTTCGTGCTGGCGCGTGGGTCCCGTT
CTTGGTTCGATCCACGGCGCTGGTGCGATCGCTGCGGATCTGGGTCCCTCGATGGTTTAGCAGCTGCGATC
GGTCAGCATCGCTGTCCCCATTCTACACTGCCATCCAAAATCAGAAAGTGTCCCCCAAGTTCGCTTGGGGG
TTCCATTGGTCTTGGCGGCATCCAGATTTGCCTGCATTCTCACGCCGTCTGCTGCTCCTTCTTCTCCTATT
TGTCTCCGTGCTGTCCGCGAATGGGTACGTTTTTCAACGTTTTGGCCTCTTGGCCATGACCCTCTTCGGTT
TCTGGATCGAACTTTGTTCGGTTATTAAGTACCTAGGTACCTACGTCTATGACTCCATCGGCCTCTCTTTC
CTCCCTTCGGACTTTTGCATATCCACAATTGGCAACTCTCACCGATCTCCATCTCGTCAACCTCCTCTCGT
GCTTTGATAACGCTTATACTATCTTTCTCTTCTCTGTTAGCATTCATTTGAAGACACTATCTTAATAAGCA
AAAACCCTAAGACGCCATCGATCTGAACAATAAAAGTCACCACGGAATTGTTCACCAAAAGTGTGAAGCAT
ATATGGCATCATCAGCCCATTGAGAGACTACTATGTAATCAAGATCAAGACTCTTCTTCCATGACTTTTGG
TGCCATTGGTCTTGGGTTGGGGGTGTAATGGTCAAATAAACGGCAAGGGGGTGGTCAGCCCTGAGCGTCGA
TGAAGAGCGTGATGATAAAAACCTATACGGTGCAAGTTGTCGATAAAGACGGGGCACATGATGGGGATTCT
TGGATGCCAGGGTCTTCCACCTAGTCCAGGGTGTCAAAGGAAATGCGGTCCCATTACTGGATCCATCCGAA
CTGCTCGAACATCGGGCCACTCCCGTTGTGTCCTCTGAGTTTCCCGATCTCAAGAGCTTCCCGATGCAGGC
TGTGATCACAGATCCTGGCCTCGGCGCTGAGACGGGATGATGGATACCGCTGTACACTAGATACGGGACAG
CAGAGCAAGCGGCACCCGGACAATGGAACAGCAGCAAGCAGTCACTCTTCAACAAAAAAAAAAATCAAAAA
ATCGAAACGGCCATGTCGCCGGCATACCACCCACAGCCTGGCTCCCGCGTTTTGTCCATGGCTCACACAAA
GACCTCCCATCTCTTGTCCCAGCCGTGGTCTCTTTGTGTACCCTCAGGCCACCCTTGCTACCTACGTACAT
GGAACGTGTAAAGGCGTCGTGTACCGTTCGATTGGATCGGCGTCTTGTCTCTGGCAACTGTCTCTCTTTTT
TTCTGTATGTCCCTGGCCCCCGTTGGCCTGCCCTGCCTGGCTCTTGAGCTATGGTGTCTCTCCATACAGG
ACCCTGGGCATCCCAAACACCCCTGGCTCCCACTTCAAAATGAACAACAACTACTACGGTTCCCCGCTGTC
TCCTGAGGAGCTTCAGCATCAAATGCACCAGCACCAGCAGCAGCAGCAGCAACAACAACAACAACAACAGC
AGCAGCAGCAGCAGCAACAACAACAACAGCAACAACAGCAACAACAGCATCAGCATCAGCAGCAACAAAAA
ACCAATCAGCATCGCAATGCCGGCATGATGAATACGCCTCCAACTACAAATCAAGGAAACAGCACGATTCA
CGCTTCAGATGTAACCATGTCAGGAGGAAGCGACTCTCTTGATGAAATCATCCAGCAGAACCTCGACGAAA
TGCATCGAAGGCGGAGTGTCCCCCAGCCCTATGGGGGCCAGACTAGGAGGTTGTCCATGTTTGATTACGCG
AACCCCAACGATGGTTTCTCGGACTATCAACTCGATAATATGTCAGGAAATTACGGGGACATGACTGGCGG
CATGGGCATGTCAGGGCACTCGAGCCCGTATGCAGGGCAGAACATCATGGCCATGTCGGACCACAGCGGTG
GATACTCGCACATGTCGCCCAATGTTATGGGCAACATGATGACTTACCCCAACCTCAACATGTACCACTCT
CCCCCGATCGAGAACCCCTATTCTTCGGCCGGACTCGACACTATTCGCACCGATTTTTCCATGGACATGAA
CATGGATAGTGGCTCCGTCAGCGCAGCCAGCGTACATCCCACCCCTGGGCTTAATAAGCAGGACGACGAGA
TGATGACCATGGAGCAGGGCTTTGGTGGAGGTGACGATGCCAACGCCTCTCACCAGGCCCAACAAAACATG
GGCGGTTTGACTCCGGCCATGACTCCGGCCATGACCCCTGCAATGACCCCGGGTGTCAGCAACTTTGCACA
AGGCATGGCTACACCAGTCTCACAGGATGCGGCAAGCACACCCGCGACAACCTTCCAGTCTCCGTCCTTAT
CCGCAACAACACAGACAATACGAATTGGGCCGCCGCCACCGCCGAGCGTCACCAATGCCCCCACCCCAGCG
CCATTTACCTCGACGCCGTCAGGTGGAGGAGCGTCCCAGACAAAGAGCATTTACTCCAAAAGCGGTTTCGA
CATGCTGAGGGCGTTGTGGTATGTGGCATCGAGGAAGGACCCAAAGCTTAAGCTCGGTGCCGTGGACATGT
CTTGTGCATTTGTCGTCTGTGATGTAACACTCAACGATTGCCCCATCATTTATGTGTCCGATAACTTCCAA
AACCTCACGGGCTATAGTCGCCACGAGATTGTCGGGCGGAACTGCCGCTTCTTGCAGGCCCCCGACGGTAA
TGTGGAGGCTGGCACCAAGCGCGAATTTGTCGAAAACAACGCCGTATATACGCTCAAGAAGACCATCGCGG
AGGGCCAGGAGATTCAGCAGAGTCTGATCAATTATCGGAAGGGCGGCAAGCCTTTCCTGAATTTGTTGACC

ATGATTCCCATTCCCTGGGATACCGAAGAAATCCGTTATTTCATTGGATTTCAGATCGATCTCGTGGAATG
CCCTGATGCCATAATCGGCCAGGAAGGAAATGGGCCCATGCAGGTCAACTATACCCACAGCGATATCGGGC
AGTACATCTGGACGCCACCTACCCAGAAGCAACTGGAGCCTGCCGACGGCCAGACTTTGGGCGTTGATGAT
GTTTCGACACTTTTGCAGCAATGTAATTCGAAGGGTGTTGCATCCGACTGGCACAAACAATCCTGGGATAA
GATGCTTTTGGAGAACGCAGACGATGTTGTCCATGTGTTGTCGCTCAAGGGTCTCTTCTTATACCTATCTC
CGGCCTGTAAGAAAGTGTTGGAGTACGATGCCAGCGACCTCGTTGGGACCTCGCTGTCCTCGATTTGCCAC
CCGTCCGACATCGTGCCGGTGACGAGGGAGTTGAAAGAGGCCCAGCAACACACTCCAGTCAATATAGTCTT
CAGAATTCGGAGAAAGAACAGTGGTTATACCTGGTTTGAAAGCCACGGAACATTGTTCAACGAACAGGGTA
AGGGTCGCAAGTGTATCATTCTTGTGGGGAGAAAACGCCCCGTTTTTGCTCTACACAGAAAGGACCTCGAA
CTCAACGGCGGTATTGGTGACAGCGAAATTTGGACCAAGGTCTCGACATCAGGCATGTTCTTGTTCGTTTC
GTCGAATGTCCGGTCGCTCCTGGATTTGCTCCCGGAGAACCTCCAAGGTACCAGCATGCAAGATCTCATGC
GCAAGGAATCCAGAGCCGAGTTTGGGAGGACGATAGAGAAAGCTAGAAAGGGCAAGATCGCGAGCTGCAAG
CATGAGGTGCAAAACAAGCGTGGCCAGGTGCTCCAGGCCTATACAACATTCTACCCCGGAGATGGTGGTGA
GGGTCAAAGGCCAACGTTTTTGTTGGCGCAAACAAAGCTGTTGAAGGCGTCATCAAGGACACTAGCTCCCG
CGACCGTAACGGTCAAGAATATGTCTCCGGGCGGTGTTCCTTTGTCGCCAATGAAAGGGATCCAGACCGAC
AGTGATAGCAACACTCTCATGGGCGGGATGTCGAAGTCGGGAAGCAGCGATAGTACGGGGGCCATGGTCTC
AGCTCGTAGCAGTGCAGGACCCGGCCAGGATGCGGCTTTGGACGCCGATAACATATTTGACGAACTCAAGA
CAACGCGTTGCACCAGCTGGCAGTACGAGCTGAGGCAAATGGAGAAGGTGAATCGCATGTTGGCCGAAGAG
CTGGCGCAGCTTTTGTCTAACAAGAAAAAACGGAAACGAAGAAAGGGAGGAGGTAACATGGTGAGGGATTG
TGCAAATTGCCATACGAGGAACACGCCCGAATGGCGGCGTGGGCCAAGCGGGAATCGAGATCTATGCAACA
GTTGTGGGTTGAGATGGGCAAAGCAGACTGGGCGCGTATCGCCGCGCACCTCATCCCGAGGTGGGAATGGT
GATTCGATGAGCAAGAAGAGCAATTCACCTAGTCACTCGTCGCCCCTTCATCGAGAAGTCGGCAACGATTC
ACCGTCTACTACCACCGCTACCAAGAACTCACCCTCTCTTCGAGGAAGTAGCACCACTGCACCCGGTACGA
TCACAACGGACAGCGGACCAGCAGTCGCATCGAGCGCTAGTGGAACCGGTAGTACGACTATAGCTACTTCA
GCCAATTCTGCTGCATCAACCGTCAACGCCCTGGGCCCTCCTGCGACAGGTCCCAGTGGCGGCTCACCGGC
TCAACATCTTCCGCCTCATCTCCAGGGAACACACCTTAACGCCCAGGCCATGCAGCGTGTACACCAGCATA
AGCAGCATCAGCAGCATCAACAGCAGCATCAACAGCAGCATCAACAGCAGCATCAACAGCAGCACCAACAG
CTTCAGCAGCATCAGTTCAACCCTCCACAAAGCCAGCCACTCCTGGAAGGCGGAAGCGGTTTCCGTGGAAG
CGGAATGGAGATGACCTCAATTAGGGAAGAGATGGGCGAACATCAACAGGGCTTAAGTGTAGGTAAGCCTA
TCCCTAACCCTCTCCTCGGTCTCGATTCTACGTAAAAGCGGCGAGTTAAGAATGGAGAGAGGAAAAGAAAA
GGGGGGAGAGCGAAAGAGAAGTGGGACGAGAGGCAACAGTCACAGCACCTCCATGGCGCAATCACAACAAC
TACAACCACAACAAGATCAACATCAACATCAACATCGACAAACAAGGGGTGGGAATCAGACAACGATGTCA
TCTGGCATACGTCGCGGCGTTTGAGGGTTTTACTTTTATGTTTGTCATTCCGGGGTTTCTCATGTTTGGTT
AAGGGGATCACATCAACATCATTGCGAAAGGCGTCTTATTCTGCGATTGGGCATGGCGATGAGATTGGTCT
TTAATTTTTTATCCTCATATTCCCCCATATATACACTACATCATTACAATCATGGGTTTCTTCACTTCCGT
CGTTCTTAAAAGACAAAGTGTTTGGTTTTTTATTTACCGCATCACCTACTCTTACATTATCATCTGTCAAG
ATGACAGAGCGGCATTCTGGCCCTCCTTTAGATCTTCATCTACAGGTTGCTTTGAATAAGGCAAATTGCAA
GAGAATTGGATCTTTCGATGAGGCGCTCTTCCATCGGCTTTTACGTTATGATGCGGGAAGCAAGCAGGCAG
GCTTGTTGGGTGGGAATGGACTGGCAGAAGCGGTAGGAGAGAGTTATGAGTCTCGCAAATCAAGGAAGGGC
CCGAGGATACAAGAGAGAGAACGCGATGACTATTGTTGCTACCGCGAGGCCACAAGGTTTTGGATTAGAAC
AGTTATTCGAGTGGGACACTGCAATGAATTAGGTCACATATCATTCTGCCAAATATGGAAACGGAATTCTG
CAAACACCGAAATATCAACAGAAGGAAATCCTATCTACTTCCATCAATGGACAAGCAGCGCTTAAACTATA
TCACTGTGCCATTGTCACACGCAACAGAGAGCGTTCATCAAAGGGCTGATCAATGATTTAAAGACGCAAAC
CGTTTGACATTCAACTTTCCTTGGACCGTCCATCATTCCGTCTAGTCCTCACTAGAGGATCCGACACTATA
GAATACCTATTCTCGGTACTATGCATTACATGTTAATTATTACATGCTTAACGTAATTCAACAGAAATTAT
ATGATAATCATCGCAAGACCGGCAACAGGATTCAATCTTAAGAAACTTTATTGCCAAATGTTTGAACGATC
TGCAGGTCGACGGATCAGATCTCGGTGACGGGCAGGACCGGACGGGGCGGTACCGGCAGGCTGAAGTCCAG
CTGCCAGAAACCCACGTCATGCCAGTTCCCGTGCTTGAAGCCGGCCGCCCGCAGCATGCCGCGGGGGGCAT
ATCCGAGCGCCTCGTGCATGCGCACGCTCGGGTCGTTGGGCAGCCCGATGACAGCGACCACGCTCTTGAAG
CCCTGTGCCTCCAGGGACTTCAGCAGGTGGGTGTAGAGCGTGGAGCCCAGTCCCGTCCGCTGGTGGCGGGG
GGAGACGTACACGGTCGACTCGGCCGTCCAGTCGTAGGCGTTGCGTGCCTTCCAGGGGCCCGCGTAGGCGA
TGCCGGCGACCTCGCCGTCCACCTCGGCGACGAGCCAGGGATAGCGCTCCCGCAGACGGACGAGGTCGTCC
GTCCACTCCTGCGGTTCCTGCGGCTCGGTACGGAAGTTGACCGTGCTTGTCTCGATGTAGTGGTTGACGAT
GGTGCAGACCGCCGGCATGTCCGCCTCGGTGGCACGGCGGATGTCGGCCGGGCGTCGTTCTGGGCTCATGG
TTACTTCCTAATCGAAGCTTTGCTTGGGTAGAATAGGTAAGTCAGATTGAATCTGAAATAAAGGGAGGAAG
GGCGAACTTAAGAAGGTATGACCGGGTCGTTCACTTACCTTGCTTGACAAACGCACCAAGTTATCGTGCAC
CAAGCAGCAGATGATAATAATGTCCTCGTTCCTGTCTGCTAATAAGAGTCACACTTCGAGCGCCGCCGCTA
CTGCTACAAGTGGGGCTGATCTGACCAGTTGCCTAAATGAACCATCTTGTCAAACGACACAAATTTTGTGC

TCACCGCCTGGACGACTAAACCAAAATAGGCATTCATTGTTGACCTCCACTAGCTCCAGCCAAGCCCAAAA
AGTGCTCCTTCAATATCATCTTCTGTCGAGTCTAGATGACTTGTTCCTGTACCTCGTCACCATCCAGCCAC
CGTCTGAAATAGACGCAGTTGCTGGGGCTGACAGGTCCGCCTTCCTCGCACATATCATAGACAGGGCACCG
CCCACAAGGCGCCTCCATAAGGCCACTTGCACTCGGCTCAACTCCCATAAATGAGGTAACAATGCCTCCTT
TGGTTGGATCATGGTCGAGGCGTCGTTGATTGCACGTCCTCGGATCCTGTTTGGGGATGCGCGTCACACGG
TACCCCTTGCGCCCATTGACCTTGACAGGCTCTACTAAGCCATCGTAGTATAGCACATCCACCAACTGCTG
GATATCCGCCTCGCTCAACGTCGTATTATTCGTGATGCCGGCGTGGTGGATGAACTTTGCAATGTCCGACA
CAGTCGGGTACGACTTGTAACCGGCCGGGAGAGGCAGCAATTGCGGCTTGCCGGGCCTGGTCTTCTTGGCC
GGTGCCGGTGCCGCCGTGTCGTCGTTGGAAATCTCGCCCGCGCTGCGCTTCGTCCCCTTGGAAATATCCTC
CGAGTGGGGAATGGTCCCCTGAACCACGCCCTTCTTGGGATTCTTTTCACGCTGCTGTGCTCCTCCTCCTT
GTGCTCCTACTCCTGGTACTCCAGCACCGCCGCCGTGGGAAGTATATGTCGATTTCTGCTTGATAAAGTCA
AACACAATTTCCTGTAGCTCGTTGATGAAGGCCGTGTCGAGCTCGCCCTCAGTAAACCAGGGGCCACCTGT
GGCGCGATCGGAGGGACGTAGGTTCGCCTTGATGTACATCTTCTTGTTGGGGTGCTCGACGTTCTTCATGC
TGGCGATGTAGCCCTTTTGCTCGAGATACTTGATGCAATGCTTCATAAGCGAGTCGGCCATGTTGAGCTTG
TTCTTGATGGTCCGGTTCCAGATGCCGTCGGCCCCGGCTTCGTCGACCATGGCGTATACCATCATTATTTC
GGAGTTGGGGAGGGAGGTGTACCTTTTGGGAGGAAGGGGAGGTTAGTTGGTGTGCTCATTTAAATGCTGTT
TCCTGTGTGAAATTGTTATCCGCTCACAATTCCACACAACATAGGAGCCGGAAGCATAAAGTGTAAAGCCT
GGGGTGCCTAATGAGTGAGGTAACTCACATTAATTGCGTTGCGCTCACTGCCCGCTTTCCAGTCGGGAAAC
CTGTCGTGCCAGCTGCATTAATGAATCGGCCAACGCGCGGGGAGAGGCGGTTTGCGTATTGGGCGCTCTTC
CGCTTCCTCGCTCACTGACTCGCTGCGCTCGGTCGTTCGGCTGCGGCGAGCGGTATCAGCTCACTCAAAGG
CGGTAATACGGTTATCCACAGAATCAGGGGATAACGCAGGAAAGAACATGTGAGCAAAAGGCCAGCAAAAG
GCCAGGAACCGTAAAAAGGCCGCGTTGCTGGCGTTTTTCCATAGGCTCCGCCCCCCTGACGAGCATCACAA
AAATCGACGCTCAAGTCAGAGGTGGCGAAACCCGACAGGACTATAAAGATACCAGGCGTTTCCCCCTGGAA
GCTCCCTCGTGCGCTCTCCTGTTCCGACCCTGCCGCTTACCGGATACCTGTCCGCCTTTCTCCCTTCGGGA
AGCGTGGCGCTTTCTCATAGCTCACGCTGTAGGTATCTCAGTTCGGTGTAGGTCGTTCGCTCCAAGCTGGG
CTGTGTGCACGAACCCCCCGTTCAGCCCGACCGCTGCGCCTTATCCGGTAACTATCGTCTTGAGTCCAACC
CGGTAAGACACGACTTATCGCCACTGGCAGCAGCCACTGGTAACAGGATTAGCAGAGCGAGGTATGTAGGC
GGTGCTACAGAGTTCTTGAAGTGGTGGCCTAACTACGGCTACACTAGAAGGACAGTATTTGGTATCTGCGC
TCTGCTGAAGCCAGTTACCTTCGGAAAAAGAGTTGGTAGCTCTTGATCCGGCAAACAAACCACCGCTGGTA
GCGGTGGTTTTTTTGTTTGCAAGCAGCAGATTACGCGCAGAAAAAAAGGATCTCAAGAAGATCCTTTGATC
TTTTCTACGGGGTCTGACGCTCAGTGGAACGAAAACTCACGTTAAGGGATTTTGGTCATGAGATTATCAAA
AAGGATCTTCACCTAGATCCTTTTAAATTAAAAATGAAGTTTTAAATCAATCTAAAGTATATATGAGTAAA
CTTGGTCTGACAGTTACCAATGCTTAATCAGTGAGGCACCTATCTCAGCGATCTGTCTATTTCGTTCATCC
ATAGTTGCCTGACTCCCCGTCGTGTAGATAACTACGATACGGGAGGGCTTACCATCTGGCCCCAGTGCTGC
AATGATACCGCGAGACCCACGCTCACCGGCTCCAGATTTATCAGCAATAAACCAGCCAGCCGGAAGGGCCG
AGCGCAGAAGTGGTCCTGCAACTTTATCCGCCTCCATCCAGTCTATTAATTGTTGCCGGGAAGCTAGAGTA
AGTAGTTCGCCAGTTAATAGTTTGCGCAACGTTGTTGCCATTGCTACAGGCATCGTGGTGTCACGCTCGTC
GTTTGGTATGGCTTCATTCAGCTCCGGTTCCCAACGATCAAGGCGAGTTACATGATCCCCCATGTTGTGCA
AAAAAGCGGTTAGCTCCTTCGGTCCTCCGATCGTTGTCAGAAGTAAGTTGGCCGCAGTGTTATCACTCATG
GTTATGGCAGCACTGCATAATTCTCTTACTGTCATGCCATCCGTAAGATGCTTTTCTGTGACTGGTGAGTA
CTCAACCAAGTCATTCTGAGAATAGTGTATGCGGCGACCGAGTTGCTCTTGCCCGGCGTCAATACGGGATA
ATACCGCGCCACATAGCAGAACTTTAAAAGTGCTCATCATTGGAAAACGTTCTTCGGGGCGAAAACTCTCA
AGGATCTTACCGCTGTTGAGATCCAGTTCGATGTAACCCACTCGTGCACCCAACTGATCTTCAGCATCTTT
TACTTTCACCAGCGTTTCTGGGTGAGCAAAAACAGGAAGGCAAAATGCCGCAAAAAAGGGAATAAGGGCGA
CACGGAAATGTTGAATACTCATACTCTTCCTTTTTCAATATTATTGAAGCATTTATCAGGGTTATTGTCTC
ATGAGCGGATACATATTTGAATGTATTTAGAAAAATAAACAAATAGGGGTTCCGCGCACATTTCCCCGAAA
AGTGCCACCTGAACGAAGCATCTGTGCTTCATTTTGTAGAACAAAAATGCAACGCGAGAGCGCTAATTTTT
CAAACAAAGAATCTGAGCTGCATTTTTACAGAACAGAAATGCAACGCGAAAGCGCTATTTTACCAACGAAG
AATCTGTGCTTCATTTTTGTAAAACAAAAATGCAACGCGAGAGCGCTAATTTTTCAAACAAAGAATCTGAG
CTGCATTTTTACAGAACAGAAATGCAACGCGAGAGCGCTATTTTACCAACAAAGAATCTATACTTCTTTTT
TGTTCTACAAAAATGCATCCCGAGAGCGCTATTTTTCTAACAAAGCATCTTAGATTACTTTTTTTCTCCTT
TGTGCGCTCTATAATGCAGTCTCTTGATAACTTTTTGCACTGTAGGTCCGTTAAGGTTAGAAGAAGGCTAC
TTTGGTGTCTATTTTCTCTTCCATAAAAAAAGCCTGACTCCACTTCCCGCGTTTACTGATTACTAGCGAAG
CTGCGGGTGCATTTTTTCAAGATAAAGGCATCCCCGATTATATTCTATACCGATGTGGATTGCGCATACTT
TGTGAACAGAAAGTGATAGCGTTGATGATTCTTCATTGGTCAGAAAATTATGAACGGTTTCTTCTATTTTG
TCTCTATATACTACGTATAGGAAATGTTTACATTTTCGTATTGTTTTCGATTCACTCTATGAATAGTTCTT
ACTACAATTTTTTTGTCTAAAGAGTAATACTAGAGATAAACATAAAAAATGTAGAGGTCGAGTTTAGATGC
AAGTTCAAGGAGCGAAAGGTGGATGGGTAGGTTATATAGGGATATAGCACAGAGATATATAGCAAAGAGAT

ACTTTTGAGCAATGTTTGTGGAAGCGGTATTCGCAATATTTTAGTAGCTCGTTACAGTCCGGTGCGTTTTT
GGTTTTTTGAAAGTGCGTCTTCAGAGCGCTTTTGGTTTTCAAAAGCGCTCTGAAGTTCCTATACTTTCTAG
AGAATAGGAACTTCGGAATAGGAACTTCAAAGCGTTTCCGAAAACGAGCGCTTCCGAAAATGCAACGCGAG
CTGCGCACATACAGCTCACTGTTCACGTCGCACCTATATCTGCGTGTTGCCTGTATATATATATACATGAG
AAGAACGGCATAGTGCGTGTTTATGCTTAAATGCGTACTTATATGCGTCTATTTATGTAGGATGAAAGGTA
GTCTAGTACCTCCTGTGATATTATCCCATTCCATGCGGGGTATCGTATGCTTCCTTCAGCACTACCCTTTA
GCTGTTCTATATGCTGCCACTCCTCAATTGGATTAGTCTCATCCTTCAATGCTATCATTTCCTTTGATATT
GGATCATACTAAGAAACCATTATTATCATGACATTAACCTATAAAAATAGGCGTATCACGAGGCCCTTTCG
TC

PVVD-GFP:
TCGCGCGTTTCGGTGATGACGGTGAAAACCTCTGACACATGCAGCTCCCGGAGACGGTCACAGCTTGTCTG
TAAGCGGATGCCGGGAGCAGACAAGCCCGTCAGGGCGCGTCAGCGGGTGTTGGCGGGTGTCGGGGCTGGCT
TAACTATGCGGCATCAGAGCAGATTGTACTGAGAGTGCACCATACCACAGCTTTTCAATTCAATTCATCAT
TTTTTTTTTATTCTTTTTTTTGATTTCGGTTTCTTTGAAATTTTTTTGATTCGGTAATCTCCGAACAGAAG
GAAGAACGAAGGAAGGAGCACAGACTTAGATTGGTATATATACGCATATGTAGTGTTGAAGAAACATGAAA
TTGCCCAGTATTCTTAACCCAACTGCACAGAACAAAAACCTGCAGGAAACGAAGATAAATCATGTCGAAAG
CTACATATAAGGAACGTGCTGCTACTCATCCTAGTCCTGTTGCTGCCAAGCTATTTAATATCATGCACGAA
AAGCAAACAAACTTGTGTGCTTCATTGGATGTTCGTACCACCAAGGAATTACTGGAGTTAGTTGAAGCATT
AGGTCCCAAAATTTGTTTACTAAAAACACATGTGGATATCTTGACTGATTTTTCCATGGAGGGCACAGTTA
AGCCGCTAAAGGCATTATCCGCCAAGTACAATTTTTTACTCTTCGAAGACAGAAAATTTGCTGACATTGGT
AATACAGTCAAATTGCAGTACTCTGCGGGTGTATACAGAATAGCAGAATGGGCAGACATTACGAATGCACA
CGGTGTGGTGGGCCCAGGTATTGTTAGCGGTTTGAAGCAGGCGGCAGAAGAAGTAACAAAGGAACCTAGAG
GCCTTTTGATGTTAGCAGAATTGTCATGCAAGGGCTCCCTATCTACTGGAGAATATACTAAGGGTACTGTT
GACATTGCGAAGAGCGACAAAGATTTTGTTATCGGCTTTATTGCTCAAAGAGACATGGGTGGAAGAGATGA
AGGTTACGATTGGTTGATTATGACACCCGGTGTGGGTTTAGATGACAAGGGAGACGCATTGGGTCAACAGT
ATAGAACCGTGGATGATGTGGTCTCTACAGGATCTGACATTATTATTGTTGGAAGAGGACTATTTGCAAAG
GGAAGGGATGCTAAGGTAGAGGGTGAACGTTACAGAAAAGCAGGCTGGGAAGCATATTTGAGAAGATGCGG
CCAGCAAAACTAAAAAACTGTATTATAAGTAAATGCATGTATACTAAACTCACAAATTAGAGCTTCAATTT
AATTATATCAGTTATTACCCTATGCGGTGTGAAATACCGCACAGATGCGTAAGGAGAAAATACCGCATCAG
GAAATTGTAAACGTTAATATTTTGTTAAAATTCGCGTTAAATTTTTGTTAAATCAGCTCATTTTTTAACCA
ATAGGCCGAAATCGGCAAAATCCCTTATAAATCAAAAGAATAGACCGAGATAGGGTTGAGTGTTGTTCCAG
TTTGGAACAAGAGTCCACTATTAAAGAACGTGGACTCCAACGTCAAAGGGCGAAAAACCGTCTATCAGGGC
GATGGCCCACTACGTGAACCATCACCCTAATCAAGTTTTTTGGGGTCGAGGTGCCGTAAAGCACTAAATCG
GAACCCTAAAGGGAGCCCCCGATTTAGAGCTTGACGGGGAAAGCCGGCGAACGTGGCGAGAAAGGAAGGGA
AGAAAGCGAAAGGAGCGGGCGCTAGGGCGCTGGCAAGTGTAGCGGTCACGCTGCGCGTAACCACCACACCC
GCCGCGCTTAATGCGCCGCTACAGGGCGCGTCGCGCCATTCGCCATTCAGGCTGCGCAACTGTTGGGAAGG
GCGATCGGTGCGGGCCTCTTCGCTATTACGCCAGCTGGCGAAAGGGGGATGTGCTGCAAGGCGATTAAGTT
GGGTAACGCCAGGGTTTTCCCAGTCACGACGATTTAAATTAGATCCAATCTGAGCCTCGCGGTGATGATTG
CCAGACGGGCGGATGCTGTGGCGACTTGGTGGTTTTTGTTCGGGCTGATCGCTTTCAGGGCGACGTTTCA
CGGTCTTGTCGGGATCAGATGGACATTGAGGTGGCCTAGCGTCAGGGTGCCGCTGAGCGCTGACCTCAACT
GACGCCGTCCAAGGCCGACCCCTGCGCTTGACCCAACACCCTGACACCAAGAAGCCAGTGGAACCCAGGAG
GTCTGCCAAGACTTTGAAAGCTTGGCACGAGCGTCTCAGCTCTTTGAAGCCTGAGTTTTCAGGCAAGAATT
CTGCTTGTAATTGCCGGGCCGGCCGACAACGCGGGGCGGCGGGAGGGGACGCTCGGCCGCCGGAGCTCGCT
TCCATGCATTTTGGATGGAGTAAAAATTGTTAAACACCCGACCCGACTCCGTTCTCCCTCTCGTCTCAGGT
CTTTGTTGTGCATCCATCGCCGTGTAGCAGATTTCCATCGTTCGTTTGTTCGGACTTTACCTTGTACTTTT
AGCTTCATTATCATCCTACAATCATCCAGGATTTCATCTTCCACAACCGGTACGTTATCATTTTTACTTTT
GTTGCCATGTTCTTCTTGAGCCTTTTCTGTCTAGAGGAGCTCTCCCTGCCGGCTGGGAGCTTGCCCAAAGC
TCCAAGCTCTATCTGTGGCAGAGCTCTGAGAGCTTCCATTCCCGGGATTTCTTGGCATCAGATTCAATTCG
AATGTCCTTTTTTCCATCCACGCTCGAGATGAGGGGTGAGGTTTGTTAGTTTGACAATACCACCACCGCCT
TTTGGTCCCTACCCCAGACATGGCCATCAAGGTCGTTAATGCCGTCCATCCCTCTTGTTCGGCTTGGGGTA
TCGTCCATGGCTCCGCAATGTCGTCAATGGCTGCATTGACAGAGGGGGACTCCAAATGAAAGAGGCAATCG
CAACGCTATGAAGACCCAACAAGCCATATGATGGAGCCATGGGTCTACGGTCCTTTGGCCCGGATGTCTTT
GGATAGCTCTTGCCAGCGCTTTAGCTGCCAACTATACACACACCGGAATTATACGATTTAGGTGACTGCAG
GATTAACCCTCACTAAAGGACAGTGGCATCAAACACAAGCCAGTGGCATCAAACACAAGCCGTCTTTAAC
ATCGGAGTATACTACACACACACGCACTCTCAGCAGTGTGTTTTTGGAAGATCGAATGCTGACTTAACACA
GGCAACCTCTTCACGAAGACCCGGCTTGCCGTTTATTATTCGGTGTAACCAAGCGCTGGGACTGTGCACCG

TGTCACATCGAGATTTCAAACTGCTCTGCGGTCGTGCAGTCACCGGTTATCATGGAACTAATTGGAAACCA
AGCGGCAGGTTGAGCAAAGTCTTGCCCTGAAAGTTGGCACATTAGAGCCATCCCGTTTGCGGTTGCAGATG
GATGAAAAACGAGCGGAATACACGGGAACAGGGCAAGGTGACCATGACATGGGCGAAAGGTAAGTAGCCGG
GGACAGACGCAACCCGCGCACATCCCGAAAGGGGAAGTTGCGATAATCCCGTGTTGTGACTTCTCCCTCTT
CCCTCGATGATGTGCCGACGCCAACGCCATCACTTATTCACGCCAGCACTGTGATTCGAGCTTGCCTGCTT
CCTGGCGTCCTATGGCCTCCCGTATCCTGCTGGTGATCATCCCAATCTGTCAAGTCTCATTTCCCCTCATT
CCCATCACGATGAGGAGCCTGAGACTTTTCGGGAAAAGAGGGGGCGTCGATAGTTTGGTCCGTTTTGTCGA
CGGTATTCATGTGATGTCCCTACAGTTGCATGAGACCAGGCCGCCCTGTTGTTGTTCATGGTTCTCTCCCA
GTCCCAAGGGAGCCAGGCTGGGGCGCCCCCCTCCCCCCCTCCTTCTTTTGTCCTTCCACAACTTCATGACC
CCCCCATGCGCCTCTGCCTAAAGGTCTACCACCTACCGGACCCTCTGCTCATCAAGACCCCCAAGATGCCC
CGCCCGCTTTATCGCCATACAGTAAATTGACTGTACATATACCCGACGATAACCTGGTTGCTGTTGGATCC
AAACTCGGGCTGTCGAGCATACAAGCGAGAAGCGTGGGTCACCTCACGGCTGGTCGGCGCAAAGGGACGAG
GTAGGTTTGTAGTGTAGCAAGGGGGTTCGGCCTTGACGCCTACAAGGGAGCAGATGGAATGGGAACACCAT
AACAACCGAGCCCAGAACCAAGGCATCACCACATGTCTTTAGCGCAACGATATCAGCAAGCTCTCCGAACA
CATATGAAGATGGGTTCCGCTATTATGGTCCTGGCGGTGTTGAGTACTTGAGGTACATATCTACCTACCTA
CCTACCTACCTACCTACCTACCTACCTACCTACCTACCTACCAACCAACCAACCTCCCCACCAACCTCTCG
GGTCGGGCAGATTAACGTTGGTGGTTAAGGTGTCAAGTGTCGTATGTCCGGTGGTGGTGGTGGTGGTGGTG
GTGCGCGGTGCGCATTTGACAGCCGGTGACGGGACAACTTGAAAGGAACCGAAACCGAAGAGACAACTTGA
CAGATGGAAGTGACTAAGTAACTCCCTAGAGCACTAGGGTTGACGTTGCAGTAAAAACGTGCCCGTTTTCT
TTCCCTATCTGCACTGTCCGCTGGCTGGTTGGTGCGGTTTCAATTGTTCGTGCTGGCGCGTGGGTCCCGTT
CTTGGTTCGATCCACGGCGCTGGTGCGATCGCTGCGGATCTGGGTCCCTCGATGGTTTAGCAGCTGCGATC
GGTCAGCATCGCTGTCCCCATTCTACACTGCCATCCAAAATCAGAAAGTGTCCCCCAAGTTCGCTTGGGGG
TTCCATTGGTCTTGGCGGCATCCAGATTTGCCTGCATTCTCACGCCGTCTGCTGCTCCTTCTTCTCCTATT
TGTCTCCGTGCTGTCCGCGAATGGGTACGTTTTTCAACGTTTTGGCCTCTTGGCCATGACCCTCTTCGGTT
TCTGGATCGAACTTTGTTCGGTTATTAAGTACCTAGGTACCTACGTCTATGACTCCATCGGCCTCTCTTTC
CTCCCTTCGGACTTTTGCATATCCACAATTGGCAACTCTCACCGATCTCCATCTCGTCAACCTCCTCTCGT
GCTTTGATAACGCTTATACTATCTTTCTCTTCTCTGTTAGCATTCATTTGAAGACACTATCTTAATAAGCA
AAAACCCTAAGACGCCATCGATCTGAACAATAAAAGTCACCACGGAATTGTTCACCAAAAGTGTGAAGCAT
ATATGGCATCATCAGCCCATTGAGAGACTACTATGTAATCAAGATCAAGACTCTTCTTCCATGACTTTTGG
TGCCATTGGTCTTGGGTTGGGGGTGTAATGGTCAAATAAACGGCAAGGGGGTGGTCAGCCCTGAGCGTCGA
TGAAGAGCGTGATGATAAAAACCTATACGGTGCAAGTTGTCGATAAAGACGGGGCACATGATGGGGATTCT
TGGATGCCAGGGTCTTCCACCTAGTCCAGGGTGTCAAAGGAAATGCGGTCCCATTACTGGATCCATCCGAA
CTGCTCGAACATCGGCCACTCCCGTTGTGTCCTCTGAGTTTCCCGATCTCAAGAGCTTCCCGATGCAGGCT
GTGATCACAGATCCTGGCCTCGGCGCTGAGACGGGATGATGGATACCGCTGTACACTAGATACGGGACAGC
AGAGCAAGCGGCACCCGGACAATGGAACAGCAGCAAGCAGTCACTCTTCAACAAAAAAAAAAATCAAAAAA
TCGAAACGGCCATGTCGCCGGCATACCACCCACAGCCTGGCTCCCGCGTTTTGTCCATGGCTCACACAAAG
ACCTCCCATCTCTTGTCCCAGCCGTGGTCTCTTTGTGTACCCTCAGGCCACCCTTGCTACCTACGTACATG
GAACGTGTAAAGGCGTCGTGTACCGTTCGATTGGATCGGCGTCTTGTCTCTGGCAACTGTCTCTCTTTTTT
TCTGTATGTCCCTGGCCCCCCGTTGGCCTGCCCTGCCTGGCTCTTGAGCTATGGTGTCTCTCCATACAGGA
CCCTGGGCATCCCAAACACCCCTGGCTCCCACTTCAAATGGTGAGCAAGGGCGAGGAGCTGTTCACCGGGG
TGGTGCCCATCCTGGTCGAGCTGGACGGCGACGTAAACGGCCACAAGTTCAGCGTGTCCGGCGAGGGCGAG
GGCGATGCCACCTACGGCAAGCTGACCCTGAAGTTCATCTGCACCACCGGCAAGCTGCCCGTGCCCTGGCC
CACCCTCGTGACCACCTTCACCTACGGCGTGCAGTGCTTCAGCCGCTACCCCGACCACATGAAGCAGCACG
ACTTCTTCAAGTCCGCCATGCCCGAAGGCTACGTCCAGGAGCGCACCATCTTCTTCAAGGACGACGGCAAC
TACAAGACCCGCGCCGAGGTGAAGTTCGAGGGCGACACCCTGGTGAACCGCATCGAGCTGAAGGGCATCGA
CTTCAAGGAGGACGGCAACATCCTGGGGCACAAGCTGGAGTACAACTACAACAGCCACAACGTCTATATCA
TGGCCGACAAGCAGAAGAACGGCATCAAGGTGAACTTCAAGATCCGCCACAACATCGAGGACGGCAGCGTG
CAGCTCGCCGACCACTACCAGCAGAACACCCCCATCGGCGACGGCCCCGTGCTGCTGCCCGACAACCACTA
CCTGAGCACCCAGTCCGCCCTGAGCAAAGACCCCAACGAGAAGCGCGATCACATGGTCCTGCTGGAGTTCG
TGACCGCCGCCGGGATCACTCTCGGCATGGACGAGCTGTACAAGGGCGGCGGAGGCGGCGGAGGCGGCGCG
CCATTAATTAACTAGAAGCGGCGAGTTAAGAATGGAGAGAGGAAAAGAAAAGGGGGGAGAGCGAAAGAGAA
GTGGGACGAGAGGCAACAGTCACAGCACCTCCATGGCGCAATCACAACAACTACAACCACAACAAGACAAC
ATCAACATCAACATCGACAAACAAGGGGTGGGAATCAGACAACGATGTCATCTGGCATACGTCGCGGCGTT
TGAGGGTTTTACTTTTATGTTTGTCATTCCGGGGTTTCTCATGTTTGGTTAAGGGGATCACATCAACATCA
TTGCGAAAGGCGTCTTATTCTGCGATTGGGCATGGCGATGAGATTGGTCTTTAATTTTTTATCCTCATATT
CCCCCATATATACACTACATCATTACAATCATGGGTTTCTTCACTTCCGTCGTTCTTAAAAGACAAAGTGT
TTGGTTTTTTATTTACCGCATCACCTACTCTTACATTATCATCTGTCAAGATGACAGAGCGGCATTCTGGC
CCTCCTTTAGATCTTCATCTACAGGTTGCTTTGAATAAGGCAAATTGCAAGAGAATTGGATCTTTCGATGA

GGCGCTCTTCCATCGGCTTTTACGTTATGATGCGGGAAGCAAGCAGGCAGGCTTGTTGGGTGGGAATGGAC
TGGCAGAAGCGGTAGGAGAGAGTTATGAGTCTCGCAAATCAAGGAAGGGCCCGAGGATACAAGAGAGAGAA
CGCGATGACTATTGTTGCTACCGCGAGGCCACAAGGTTTTGGATTAGAACAGTTATTCGAGTGGGACACTG
CAATGAATTAGGTCACATATCATTCTGCCAAATATGGAAACGGAATTCTGCAAACACCGAAATATCAACAG
AAGGAAATCCTATCTACTTCCATCAATGGACAAGCAGCGCTTAAACTATATCACTGTGCCATTGTCACACG
CAACAGAGAGCGTTCATCAAAGGGCTGATCAATGATTTAAAGACGCAAACCGTTTGACATTCAACTTTCCT
TGGACCGTCCATCATTCCGTCTAGTCCTCACTAGAGGATCCGACACTATAGAATACCTATTCTCGGTACTA
TGCATTACATGTTAATTATTACATGCTTAACGTAATTCAACAGAAATTATATGATAATCATCGCAAGACCG
GCAACAGGATTCAATCTTAAGAAACTTTATTGCCAAATGTTTGAACGATCTGCAGGTCGACGGATCAGATC
TCGGTGACGGGCAGGACCGGACGGGGCGGTACCGGCAGGCTGAAGTCCAGCTGCCAGAAACCCACGTCATG
CCAGTTCCCGTGCTTGAAGCCGGCCGCCCGCAGCATGCCGCGGGGGGCATATCCGAGCGCCTCGTGCATGC
GCACGCTCGGGTCGTTGGGCAGCCCGATGACAGCGACCACGCTCTTGAAGCCCTGTGCCTCCAGGGACTTC
AGCAGGTGGGTGTAGAGCGTGGAGCCCAGTCCCGTCCGCTGGTGGCGGGGGGAGACGTACACGGTCGACTC
GGCCGTCCAGTCGTAGGCGTTGCGTGCCTTCCAGGGGCCCGCGTAGGCGATGCCGGCGACCTCGCCGTCCA
CCTCGGCGACGAGCCAGGGATAGCGCTCCCGCAGACGGACGAGGTCGTCCGTCCACTCCTGCGGTTCCTGC
GGCTCGGTACGGAAGTTGACCGTGCTTGTCTCGATGTAGTGGTTGACGATGGTGCAGACCGCCGGCATGTC
CGCCTCGGTGGCACGGCGGATGTCGGCCGGGCGTCGTTCTGGGCTCATGGTTACTTCCTAATCGAAGCTTT
GCTTGGGTAGAATAGGTAAGTCAGATTGAATCTGAAATAAAGGGAGGAAGGGCGAACTTAAGAAGGTATGA
CCGGGTCGTTCACTTACCTTGCTTGACAAACGCACCAAGTTATCGTGCACCAAGCAGCAGATGATAATAAT
GTCCTCGTTCCTGTCTGCTAATAAGAGTCACACTTCGAGCGCCGCCGCTACTGCTACAAGTGGGGCTGATC
TGACCAGTTGCCTAAATGAACCATCTTGTCAAACGACACAAATTTTGTGCTCACCGCCTGGACGACTAAAC
CAAAATAGGCATTCATTGTTGACCTCCACTAGCTCCAGCCAAGCCCAAAAAGTGCTCCTTCAATATCATCT
TCTGTCGAGTCTAGATGACTTGTTCCTGTACCTCGTCACCATCCAGCCACCGTCTGAAATAGACGCAGTTG
CTGGGGCTGACAGGTCCGCCTTCCTCGCACATATCATAGACAGGGCACCGCCCACAAGGCGCCTCCATAAG
GCCACTTGCACTCGGCTCAACTCCCATAAATGAGGTAACAATGCCTCCTTTGGTTGGATCATGGTCGAGGC
GTCGTTGATTGCACGTCCTCGGATCCTGTTTGGGGATGCGCGTCACACGGTACCCCTTGCGCCCATTGACC
TTGACAGGCTCTACTAAGCCATCGTAGTATAGCACATCCACCAACTGCTGGATATCCGCCTCGCTCAACGT
CGTATTATTCGTGATGCCGGCGTGGTGGATGAACTTTGCAATGTCCGACACAGTCGGGTACGACTTGTAAC
CGGCCGGGAGAGGCAGCAATTGCGGCTTGCCGGGCCTGGTCTTCTTGGCCGGTGCCGGTGCCGCCGTGTCG
TCGTTGGAAATCTCGCCCGCGCTGCGCTTCGTCCCCTTGGAAATATCCTCCGAGTGGGGAATGGTCCCCTG
AACCACGCCCTTCTTGGGATTCTTTTCACGCTGCTGTGCTCCTCCTCCTTGTGCTCCTACTCCTGGTACTC
CAGCACCGCCGCCGTGGGAAGTATATGTCGATTTCTGCTTGATAAAGTCAAACACAATTTCCTGTAGCTCG
TTGATGAAGGCCGTGTCGAGCTCGCCCTCAGTAAACCAGGGGCCACCTGTGGCGCGATCGGAGGGACGTAG
GTTCGCCTTGATGTACATCTTCTTGTTGGGGTGCTCGACGTTCTTCATGCTGGCGATGTAGCCCTTTTGCT
CGAGATACTTGATGCAATGCTTCATAAGCGAGTCGGCCATGTTGAGCTTGTTCTTGATGGTCCGGTTCCAG
ATGCCGTCGGCCCCGGCTTCGTCGACCATGGCGTATACCATCATTATTTCGGAGTTGGGGAGGGAGGTGTA
CCTTTTGGGAGGAAGGGGAGGTTAGTTGGTGTGCTCATTTAAATGCTGTTTCCTGTGTGAAATTGTTATCC
GCTCACAATTCCACACAACATAGGAGCCGGAAGCATAAAGTGTAAAGCCTGGGGTGCCTAATGAGTGAGGT
AACTCACATTAATTGCGTTGCGCTCACTGCCCGCTTTCCAGTCGGGAAACCTGTCGTGCCAGCTGCATTAA
TGAATCGGCCAACGCGCGGGGAGAGGCGGTTTGCGTATTGGGCGCTCTTCCGCTTCCTCGCTCACTGACTC
GCTGCGCTCGGTCGTTCGGCTGCGGCGAGCGGTATCAGCTCACTCAAAGGCGGTAATACGGTTATCCACAG
AATCAGGGGATAACGCAGGAAAGAACATGTGAGCAAAAGGCCAGCAAAAGGCCAGGAACCGTAAAAAGGCC
GCGTTGCTGGCGTTTTTCCATAGGCTCCGCCCCCCTGACGAGCATCACAAAAATCGACGCTCAAGTCAGAG
GTGGCGAAACCCGACAGGACTATAAAGATACCAGGCGTTTCCCCCTGGAAGCTCCCTCGTGCGCTCTCCTG
TTCCGACCCTGCCGCTTACCGGATACCTGTCCGCCTTTCTCCCTTCGGGAAGCGTGGCGCTTTCTCATAGC
TCACGCTGTAGGTATCTCAGTTCGGTGTAGGTCGTTCGCTCCAAGCTGGGCTGTGTGCACGAACCCCCCGT
TCAGCCCGACCGCTGCGCCTTATCCGGTAACTATCGTCTTGAGTCCAACCCGGTAAGACACGACTTATCGC
CACTGGCAGCAGCCACTGGTAACAGGATTAGCAGAGCGAGGTATGTAGGCGGTGCTACAGAGTTCTTGAAG
TGGTGGCCTAACTACGGCTACACTAGAAGGACAGTATTTGGTATCTGCGCTCTGCTGAAGCCAGTTACCTT
CGGAAAAAGAGTTGGTAGCTCTTGATCCGGCAAACAAACCACCGCTGGTAGCGGTGGTTTTTTTGTTTGCA
AGCAGCAGATTACGCGCAGAAAAAAAGGATCTCAAGAAGATCCTTTGATCTTTTCTACGGGGTCTGACGCT
CAGTGGAACGAAAACTCACGTTAAGGGATTTTGGTCATGAGATTATCAAAAAGGATCTTCACCTAGATCCT
TTTAAATTAAAAATGAAGTTTTAAATCAATCTAAAGTATATATGAGTAAACTTGGTCTGACAGTTACCAAT
GCTTAATCAGTGAGGCACCTATCTCAGCGATCTGTCTATTTCGTTCATCCATAGTTGCCTGACTCCCCGTC
GTGTAGATAACTACGATACGGGAGGGCTTACCATCTGGCCCCAGTGCTGCAATGATACCGCGAGACCCACG
CTCACCGGCTCCAGATTTATCAGCAATAAACCAGCCAGCCGGAAGGGCCGAGCGCAGAAGTGGTCCTGCAA
CTTTATCCGCCTCCATCCAGTCTATTAATTGTTGCCGGGAAGCTAGAGTAAGTAGTTCGCCAGTTAATAGT
TTGCGCAACGTTGTTGCCATTGCTACAGGCATCGTGGTGTCACGCTCGTCGTTTGGTATGGCTTCATTCAG

CTCCGGTTCCCAACGATCAAGGCGAGTTACATGATCCCCCATGTTGTGCAAAAAAGCGGTTAGCTCCTTCG
GTCCTCCGATCGTTGTCAGAAGTAAGTTGGCCGCAGTGTTATCACTCATGGTTATGGCAGCACTGCATAAT
TCTCTTACTGTCATGCCATCCGTAAGATGCTTTTCTGTGACTGGTGAGTACTCAACCAAGTCATTCTGAGA
ATAGTGTATGCGGCGACCGAGTTGCTCTTGCCCGGCGTCAATACGGGATAATACCGCGCCACATAGCAGAA
CTTTAAAAGTGCTCATCATTGGAAAACGTTCTTCGGGGCGAAAACTCTCAAGGATCTTACCGCTGTTGAGA
TCCAGTTCGATGTAACCCACTCGTGCACCCAACTGATCTTCAGCATCTTTTACTTTCACCAGCGTTTCTGG
GTGAGCAAAAACAGGAAGGCAAAATGCCGCAAAAAAGGGAATAAGGGCGACACGGAAATGTTGAATACTCA
TACTCTTCCTTTTTCAATATTATTGAAGCATTTATCAGGGTTATTGTCTCATGAGCGGATACATATTTGAA
TGTATTTAGAAAAATAAACAAATAGGGGTTCCGCGCACATTTCCCCGAAAAGTGCCACCTGAACGAAGCAT
CTGTGCTTCATTTTGTAGAACAAAAATGCAACGCGAGAGCGCTAATTTTTCAAACAAAGAATCTGAGCTGC
ATTTTTACAGAACAGAAATGCAACGCGAAAGCGCTATTTTACCAACGAAGAATCTGTGCTTCATTTTTGTA
AAACAAAAATGCAACGCGAGAGCGCTAATTTTTCAAACAAAGAATCTGAGCTGCATTTTTACAGAACAGAA
ATGCAACGCGAGAGCGCTATTTTACCAACAAAGAATCTATACTTCTTTTTTGTTCTACAAAAATGCATCCC
GAGAGCGCTATTTTTCTAACAAAGCATCTTAGATTACTTTTTTTCTCCTTTGTGCGCTCTATAATGCAGTC
TCTTGATAACTTTTTGCACTGTAGGTCCGTTAAGGTTAGAAGAAGGCTACTTTGGTGTCTATTTTCTCTTC
CATAAAAAAAGCCTGACTCCACTTCCCGCGTTTACTGATTACTAGCGAAGCTGCGGGTGCATTTTTTCAAG
ATAAAGGCATCCCCGATTATATTCTATACCGATGTGGATTGCGCATACTTTGTGAACAGAAAGTGATAGCG
TTGATGATTCTTCATTGGTCAGAAAATTATGAACGGTTTCTTCTATTTTGTCTCTATATACTACGTATAGG
AAATGTTTACATTTTCGTATTGTTTTCGATTCACTCTATGAATAGTTCTTACTACAATTTTTTTGTCTAAA
GAGTAATACTAGAGATAAACATAAAAAATGTAGAGGTCGAGTTTAGATGCAAGTTCAAGGAGCGAAAGGTG
GATGGGTAGGTTATATAGGGATATAGCACAGAGATATATAGCAAAGAGATACTTTTGAGCAATGTTTGTGG
AAGCGGTATTCGCAATATTTTAGTAGCTCGTTACAGTCCGGTGCGTTTTTGGTTTTTTGAAAGTGCGTCTT
CAGAGCGCTTTTGGTTTTCAAAAGCGCTCTGAAGTTCCTATACTTTCTAGAGAATAGGAACTTCGGAATAG
GAACTTCAAAGCGTTTCCGAAAACGAGCGCTTCCGAAAATGCAACGCGAGCTGCGCACATACAGCTCACTG
TTCACGTCGCACCTATATCTGCGTGTTGCCTGTATATATATATACATGAGAAGAACGGCATAGTGCGTGTT
TATGCTTAAATGCGTACTTATATGCGTCTATTTATGTAGGATGAAAGGTAGTCTAGTACCTCCTGTGATAT
TATCCCATTCCATGCGGGGTATCGTATGCTTCCTTCAGCACTACCCTTTAGCTGTTCTATATGCTGCCACT
CCTCAATTGGATTAGTCTCATCCTTCAATGCTATCATTTCCTTTGATATTGGATCATACTAAGAAACCATT
ATTATCATGACATTAACCTATAAAAATAGGCGTATCACGAGGCCCTTTCGTC

PRIMERS:
WC-1 FORWARD
GAAGCGGAATGGAGATGAC

WC-1 REVERSE
ATTCTTAACTCGCCGCTTCT

GFP FORWARD
TGACCCTGAAGTTCATCTGC

GFP REVERSE
GAAGTCGTGCTGCTTCATGT

ACTIN FORWARD
TCCATCATGAAGTGCGATGT

ACTIN REVERSE
CCTTCATGGAAGAAGGAGCA
